# Targeting Toll‐like receptor‐4 to tackle preterm birth and fetal inflammatory injury

**DOI:** 10.1002/cti2.1121

**Published:** 2020-04-14

**Authors:** Sarah A Robertson, Mark R Hutchinson, Kenner C Rice, Peck‐Yin Chin, Lachlan M Moldenhauer, Michael J Stark, David M Olson, Jeffrey A Keelan

**Affiliations:** ^1^ Robinson Research Institute and Adelaide Medical School University of Adelaide Adelaide SA Australia; ^2^ ARC Centre for Nanoscale Biophotonics and Adelaide Medical School University of Adelaide Adelaide SA Australia; ^3^ Drug Design and Synthesis Section National Institute on Drug Abuse and National Institute on Alcohol Abuse and Alcoholism National Institutes of Health Rockville MD USA; ^4^ Department of Obstetrics and Gynecology Department of Physiology and Pediatrics 220 HMRC University of Alberta Edmonton AB Canada; ^5^ Division of Obstetrics & Gynaecology University of Western Australia Perth WA Australia

**Keywords:** fetal inflammatory injury, inflammation, pregnancy, preterm, TLR4 antagonist, Toll‐like receptor 4

## Abstract

Every year, 15 million pregnancies end prematurely, resulting in more than 1 million infant deaths and long‐term health consequences for many children. The physiological processes of labour and birth involve essential roles for immune cells and pro‐inflammatory cytokines in gestational tissues. There is compelling evidence that the mechanisms underlying spontaneous preterm birth are initiated when a premature and excessive inflammatory response is triggered by infection or other causes. Exposure to pro‐inflammatory mediators is emerging as a major factor in the ‘fetal inflammatory response syndrome’ that often accompanies preterm birth, where unscheduled effects in fetal tissues interfere with normal development and predispose to neonatal morbidity. Toll‐like receptors (TLRs) are critical upstream gatekeepers of inflammatory activation. TLR4 is prominently involved through its ability to sense and integrate signals from a range of microbial and endogenous triggers to provoke and perpetuate inflammation. Preclinical studies have identified TLR4 as an attractive pharmacological target to promote uterine quiescence and protect the fetus from inflammatory injury. Novel small‐molecule inhibitors of TLR4 signalling, specifically the non‐opioid receptor antagonists (+)‐naloxone and (+)‐naltrexone, are proving highly effective in animal models for preventing preterm birth induced by bacterial mimetic LPS, heat‐killed *Escherichia coli*, or the TLR4‐dependent pro‐inflammatory lipid, platelet‐activating factor (PAF). Here, we summarise the rationale for targeting TLR4 as a master regulator of inflammation in fetal and gestational tissues, and the potential utility of TLR4 antagonists as candidates for preventative and therapeutic application in preterm delivery and fetal inflammatory injury.

## Graphical abstract

Inflammation is the central mechanism of spontaneous preterm birth, and TLR4 is an essential mediator of the microbial and endogenous pro‐inflammatory triggers. Preclinical studies identify TLR4 as an attractive pharmacological target to protect against preterm birth and fetal inflammatory injury. In this review, we discuss the biological rationale and clinical potential of novel small‐molecule inhibitors of TLR4 signalling that offer promise for preventative and therapeutic applications in preterm birth.

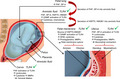

## Introduction

New therapeutic options to tackle spontaneous preterm birth and mitigate its adverse impact in infants born prematurely are urgently needed.^1^, ^2^ Innate immune activation leading to inflammation in gestational tissues is a central, early and rate‐limiting mechanism driving preterm birth.^3^, ^4^ Upstream events in the inflammatory mechanisms that elicit fetal and placental stress and precede active labour are attractive targets for intervention.^5^, ^6^, ^7^ To progress pharmacological solutions, the complex signals involved in initiating parturition, and the mechanisms by which different triggers converge onto a common inflammatory cascade, must be defined.^4^

**Figure 1:**
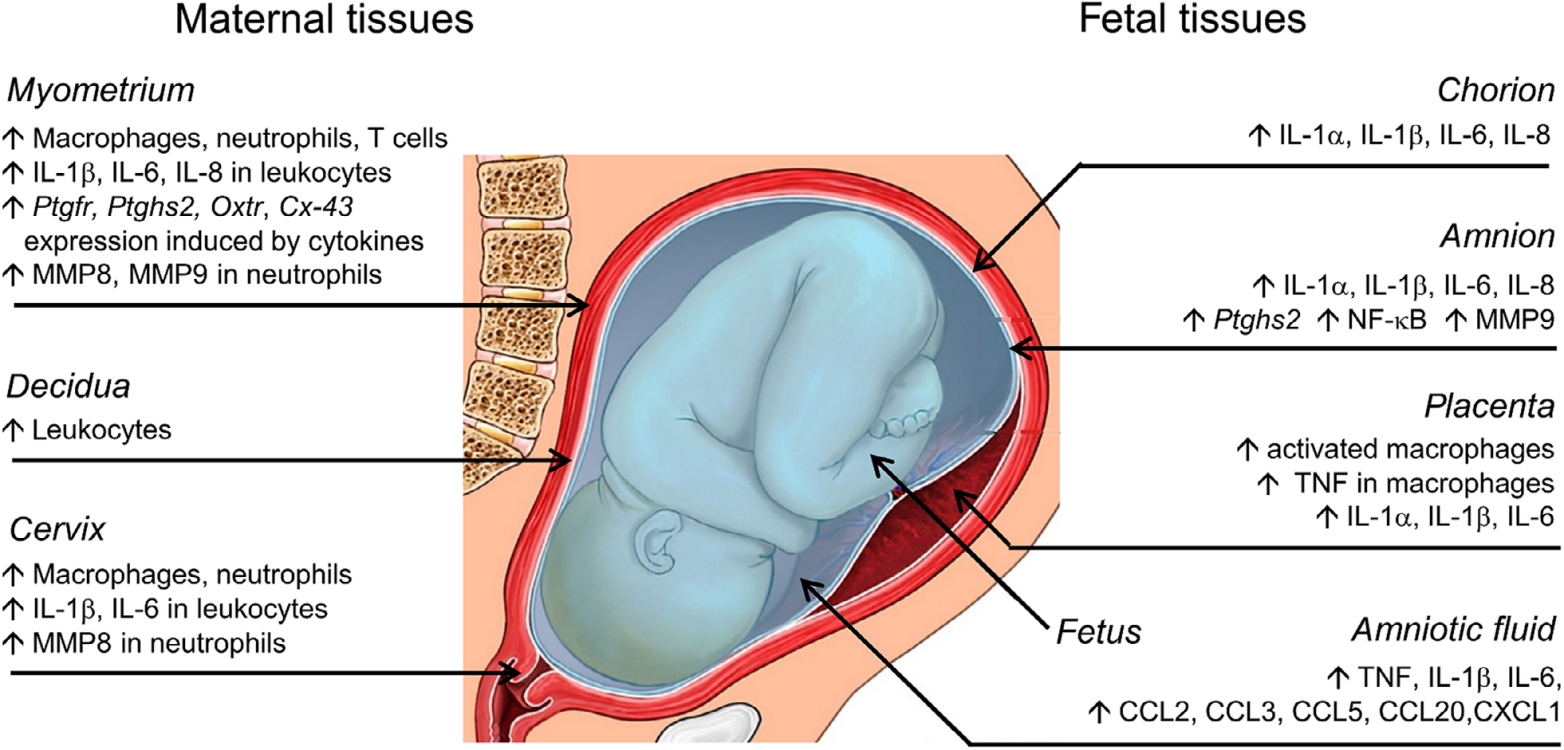
Indicators of inflammatory activation in fetal and maternal tissues during preterm labour. Inflammatory activation is central to parturition, with molecular and cellular changes that manifest in the fetal and maternal compartments. In maternal tissues including the myometrium, decidua and cervix, recruitment of inflammatory leucocytes and elevated expression of pro‐inflammatory cytokines and chemokines are evident. These pro‐inflammatory mediators upregulate uterine activation genes, in turn causing myometrial contractions and cervical effacement and dilation. Macrophages infiltrate the placenta and cause elevated production of pro‐inflammatory cytokines. The fetal membranes (amnion and chorion) express elevated inflammatory cytokines, which access uterine tissues to promote uterine activation gene expression and amplify MMP production, instigating fetal membrane rupture. In the amniotic fluid, elevated accumulation of inflammatory cytokines and chemokines may be transmitted to the fetal and maternal tissues. The underlying pro‐inflammatory drivers in preterm labour are a consequence of infection, or sterile tissue insult or injury.

Infection is a common cause of preterm birth, but sterile factors and insults, such as exposure to oxidative stress and toxins, immune or endocrine imbalance, multiple births, and placental hypoxia and haemorrhage, are also risk factors.^4^, ^8^ For both microbial and sterile causes, inflammatory activation occurs early in the pathophysiological pathway.^3^, ^9^, ^10^ Pro‐inflammatory cytokines and effector molecules are produced in the fetal membranes, myometrium and cervix well before uterine contractions, membrane rupture and cervical dilatation occur.^9^, ^10^, ^11^ These tissue changes are accompanied by extensive accumulation of leucocytes from both the innate and adaptive immune compartments. Leucocytes progressively infiltrate the uterine myometrium, decidua and fetal membranes, along with elevated expression of cytokines and chemokines consistent with a pro‐inflammatory profile, in the days and weeks ahead of the final delivery phase.^12^, ^13^, ^14^, ^15^ These activated immune cells and their cytokine mediators in turn erode the local anti‐inflammatory mechanisms of pregnancy tolerance provided by progesterone and regulatory T (Treg) cells.^12^, ^16^, ^17^ They also promote elevated synthesis of pro‐labour ‘uterine activation genes’ (UAGs) encoding prostaglandins and tissue‐remodelling enzymes that override uterine quiescence and drive progression to birth^9^, ^10^ (Figure 1).

The inflammatory processes of term and preterm birth are broadly comparable, but in preterm birth there can be different initiating triggers, kinetics and scale of response, and a lack of coordinating regulation.^10^, ^18^ For immature fetal tissues and organs, premature exposure to high levels of pro‐inflammatory effectors can perturb development. In particular, the immature fetal lungs, gastrointestinal tract, brain and heart are susceptible to damage, resulting in health complications after birth with long‐lasting consequences.^19^ The severity of this ‘fetal inflammatory response syndrome’ is worse for babies born at lower gestational age.^20^ Early preterm infants are at high risk of cerebral palsy, neurocognitive dysfunction, and respiratory and gastrointestinal complications.^19^, ^20^ Even late preterm infants have an elevated risk of chronic diseases such as obesity, hypertension and diabetes in adult life.^1^

**Figure 2:**
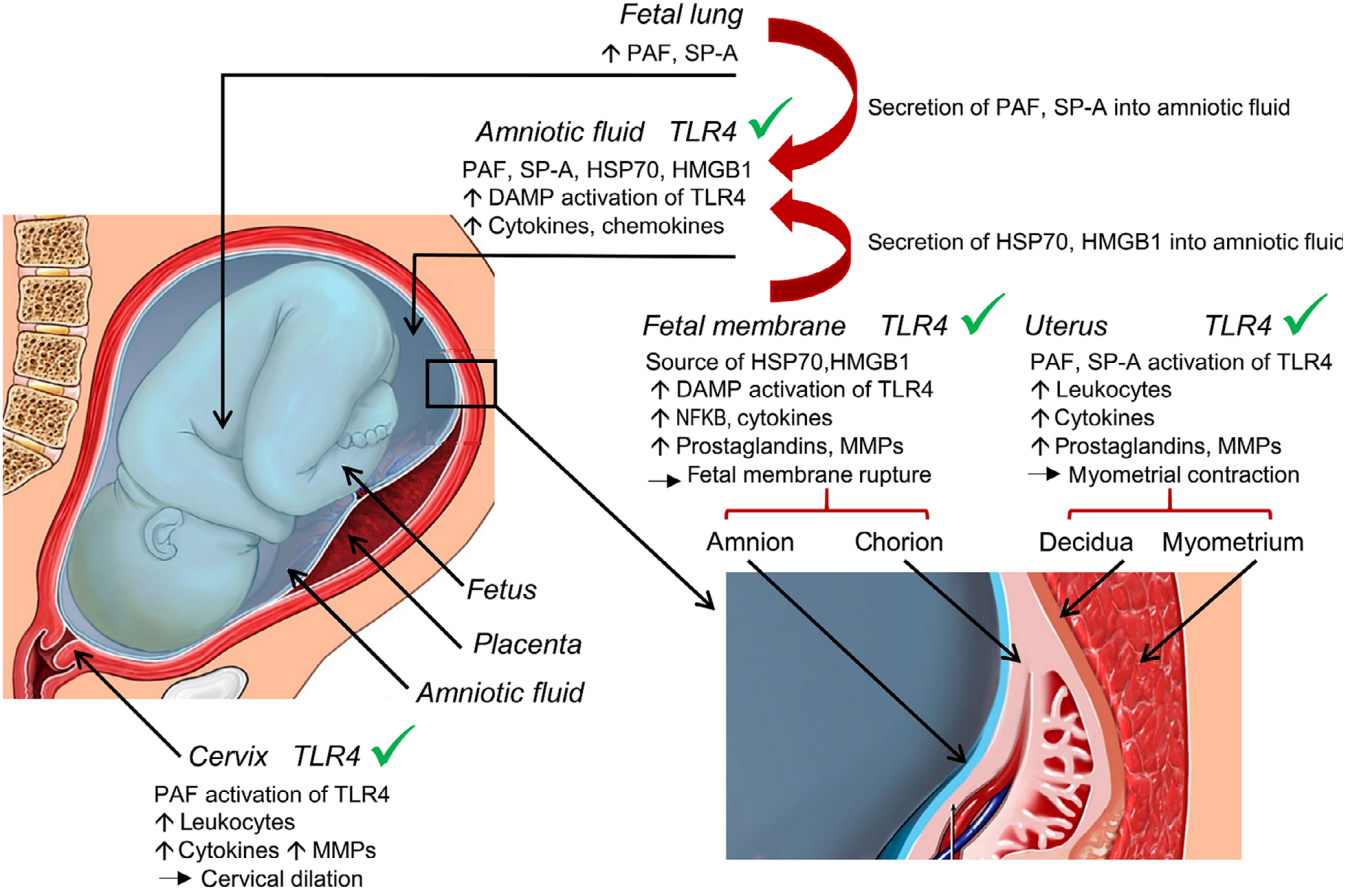
Damage‐associated molecular patterns (DAMPs), pathogen‐associated molecular patterns (PAMPs) and endogenous TLR4 activators in gestational tissues during preterm labour. An array of TLR4 ligands and activators accumulate in fetal and maternal tissues where they drive an amplifying inflammatory cascade of cytokine expression and leucocyte infiltration. TLR4 ligands including LPS and other PAMPs of microbial origin are produced by microbial infection. Endogenous DAMPs produced during sterile tissue insult or injury can also activate TLR4. These DAMPs include HSP70 and HMGB1 released from fetal membranes and PAF and SP‐A released from fetal lungs. DAMPs can also be released after microbial infection. TLR4 is abundantly expressed by leucocytes and other cell lineages in fetal membranes, uterine decidua and myometrium, and cervical tissues. TLR4 ligands can be transmitted from amniotic fluid into the myometrium and cervix, to amplify inflammatory activation and ultimately cause uterine contractions, cervical dilation and delivery of the fetus.

Toll‐like receptors (TLRs) are pivotal upstream gatekeepers of innate immune activation and are abundantly expressed in the placenta, fetal membranes and uterus.^21^, ^22^ In particular, TLR4 has been identified as a key regulator of the inflammatory processes that control normal on‐time birth,^23^ and premature activation of TLR4 signalling can provoke preterm delivery. Premature induction of TLR4 signalling in several compartments of the gestational tissues stimulates pro‐inflammatory cytokine and chemokine expression and leucocyte infiltration (Figure 2) that becomes amplified through loops of feed‐forward mechanisms to initiate uterine transformation and drive progression to preterm labour. Since TLR4 is a promiscuous sensor of both microbial and sterile pro‐inflammatory signals in gestational tissues,^5^, ^24^ including endogenous agents released upon cell senescence or death after injury or infection,^25^ it has the potential to act as a point of convergence through which microbial and sterile agents all trigger preterm labour. From an evolutionary perspective, the potential of TLR4 to sense and integrate signals of fetal organ maturation, infection, and tissue damage and senescence confers the benefit of initiating birth when *in utero* conditions are unfavorable for continued fetal development and viability. Substantial clinical data point to a critical role for TLR4 and innate immune activation in humans – a large genome‐wide association study (> 40 000 women) identifies rare variants in genes encoding negative regulators of innate immunity and anti‐microbial defence, as predisposing to preterm birth.^26^

In this review, we assemble evidence from animal models and clinical studies implicating TLR4 as a key rate‐limiting mediator in preterm birth and discuss novel compounds that inhibit TLR4 signalling and their potential utility in suppressing inflammation to extend gestation, and protect the fetus from inflammatory injury.

## TLR4 and innate immune activation

TLR4 is one of 13 receptors, each with specific sets of cognate ligands, which make up the mammalian TLR system. Each TLR engages ligands released by different microbes, known as pathogen‐associated molecular patterns (PAMPs). The key microbial ligand for TLR4 is lipopolysaccharide (LPS, endotoxin) from cell walls of Gram‐negative bacteria. TLR2 recognises peptidoglycan (PGN) and lipoteichoic acid (LTA) common to several bacterial taxa, while other TLRs bind double‐stranded RNA viral motifs or bacterial flagellin.^27^ TLR signal transduction is complex and is regulated by bioavailability of various proteins and co‐receptors.^27^ TLR4 is not directly ligated by products of Gram‐positive bacteria, but can amplify an inflammatory response initiated by TLR2.^28^

**Figure 3:**
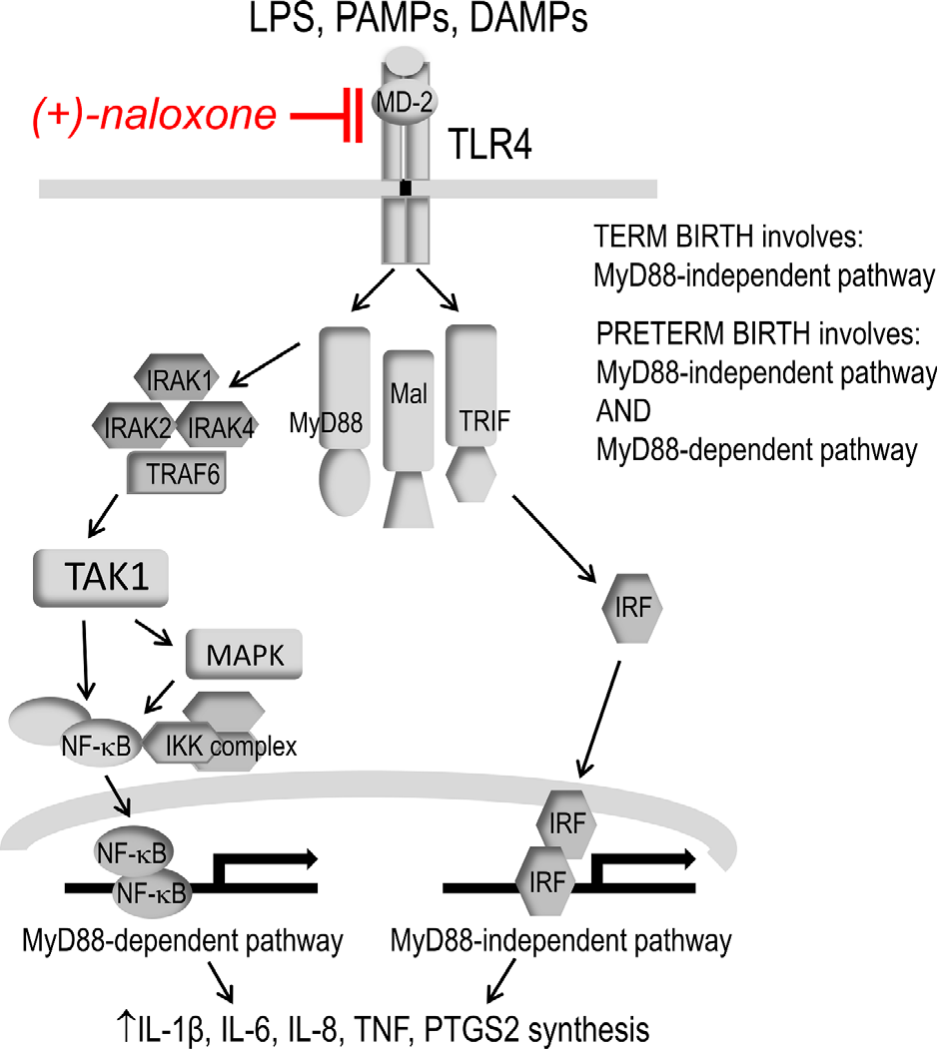
Schematic illustration of the MyD88‐dependent and MyD88‐independent pathways mediated by TLR4 ligation to induce NF‐κB activation and cytokine gene expression. TLR4 activates the NF‐κB transcription factor via the adaptor molecules MyD88 and Mal, which activate several kinases including TAK1 in the MyD88‐dependent pathway. The IRF transcription factor is activated by TLR4 via TRIF, an alternative adaptor molecule in the MyD88‐independent pathway. The TLR signalling antagonist (+)‐naloxone binds MD‐2 to prevent TLR4 engaging with LPS or other ligands. Term labour is mediated by the MyD88‐independent but not the MyD88‐dependent pathway of TLR4 signalling, while preterm birth involves both signalling pathways (see text for details)

Typically, LPS activation of TLR4 signalling involves formation of a receptor complex consisting of TLR4, MD‐2 and adaptor molecules including myeloid differentiation factor 88 (MyD88) and MyD88 adaptor‐like (Mal), as well as TIR domain‐containing adaptor‐inducing interferon‐beta (TRIF)^25^, ^27^ (Figure 3). The MyD88‐dependent pathway stimulates activation of TGF‐β‐associated kinase (TAK)‐1, interleukin‐1 receptor (IL‐1R)‐associated kinases IRAK1 and IRAK4, TRF‐associated factor 6 (TRAF6) and mitogen‐activated kinases (MAPK), which in turn activate NF‐κB via the IκB kinase (IKK) complex, to initiate transcription of genes encoding IL‐1β, IL‐6, TNF and other pro‐inflammatory cytokines.^25^, ^27^ A MyD88‐independent pathway is also initiated after TRIF‐induced activation of the interferon‐regulated factor (IRF) family of transcription factors, to mediate the transcription of type 1 interferons (IFN)^25^, ^27^(Figure 3). There is extensive cross‐regulation between the TLRs controlled by integrated regulatory interactions at the level of receptor, adaptors, signalling molecules and transcription factors, as well as attenuation by microRNAs. This regulatory network is modifiable through ‘innate immune memory’, whereby previous exposures to inflammatory activation can programme elevated tolerance, or higher sensitivity, to subsequent inflammatory activation.^29^ The significance of innate immune memory in susceptibility to preterm birth is yet to be explored.^10^

As well as microbial elements, TLRs recognise endogenously produced agents released from intracellular and extracellular compartments after cell stress or necrotic cell death. Endogenous TLR ligands are known as damage‐associated molecular patterns (DAMPs, or ‘alarmins’). DAMPs provide a physiological signal of tissue stress and damage, to initiate tissue defence and repair mechanisms.^25^, ^30^ When released at low levels, DAMPs are important for modulating a physiological immune response to regain tissue homeostasis.^25^, ^30^ After tissue injury or in chronic pathological situations, DAMP release increases, and overt and persistent inflammation can result. There are synergistic interactions between DAMPs and PAMPs, such that DAMPs released after infection‐induced tissue damage act to amplify the inflammatory response beyond levels caused by microbial products alone.

## TLR4 signalling and the labour cascade

An array of TLRs is expressed in a spatially and temporally controlled manner in the fetal and maternal compartments of the gestational tissues. TLR4 is strongly expressed in the fetal membranes, placental trophoblasts and endocervix^22^, ^31^, ^32^ and is prominent on resident immune cells including uterine NK cells, macrophages and neutrophils.^33^, ^34^ At term, responsiveness to TLR4 signalling is elevated in the chorioamniotic membrane when increased transcription of *TLR4* occurs.^31^

Mouse models have been instrumental in defining TLR4 as a rate‐limiting effector at the apex of the inflammatory response driving uterine activation and controlling timing of labour. In mice, TLR4 is readily detectable in maternal tissues including uterine decidua and cervix, fetal membranes and placenta, and becomes elevated in the cervix and uterus towards the end of gestation.^35^, ^36^ TLR4‐deficient (*Tlr4* ^−/−^) mice have an extended gestation length and increased perinatal mortality compared to wild‐type controls.^23^ This is associated with disrupted expression of pro‐inflammatory cytokines *Il1b*, *I16*, *I112b* and *Tnf* normally induced in wild‐type fetal membranes, placenta and uterus prior to term labour. Additionally, *Tlr4* ^−/−^ mice have delayed expression of UAGs involved in transitioning the uterus from a quiescent to contractile state, notably prostaglandin F receptor, oxytocin receptor and connexin‐43. Leucocyte accumulation is impaired in TLR4‐deficient females, with fewer neutrophils and macrophages in the placenta, and fewer dendritic cells and more regulatory T cells in the uterus, compared to wild‐type mice. Unlike TLR4 deficiency, genetic disruption of MyD88 does not delay parturition,^23^ presumably since NF‐κB activation can occur without MyD88 and pro‐inflammatory cytokines are induced via both MyD88 and TRIF‐dependent signalling.^37^

TLR2 interacts with TLR4 to regulate labour, and there is redundancy between the two receptors. Mice with a null mutation in *Tlr2* (*Tlr2* ^−/−^ mice) have delayed labour compared to wild‐type mice,^38^ accompanied by delayed induction of UAGs and reduced myometrial macrophage accumulation.^38^ Additionally, amniotic fluid macrophages isolated from *Tlr2* ^−/−^ mice in late gestation exhibit altered expression of activation markers *Il1b* and *Arg1* mRNA.^38^

Since infection is not usually present in healthy term labour, endogenous DAMPs including HMGB1, cell‐free DNA and oxysterols released from senescent cells in fetal membranes and placenta are implicated in TLR4 signalling in term labour. There is compelling evidence that pro‐inflammatory signals released from the maturing fetal lung, notably surfactant protein‐A (SP‐A), SP‐D and PAF, drive inflammation in fetal membranes and uterine tissues to precipitate the labour cascade.^39^ These agents likely provoke fetal membrane cell stress and release of DAMPs that amplify local inflammation through a TLR4‐dependent cascade (see later).

## Infectious and sterile triggers of preterm birth

In preterm labour, TLR4 ligands accumulate prematurely as a result of infection and/or sterile stressors in maternal and/or fetal compartments in the gestational tissues. Intrauterine infection occurs in 25–40% of preterm birth cases, particularly in early preterm birth.^8^ In the event of ascending uterine infection, bacteria progressively infiltrate the decidua, chorion and amnion; then, ultimately microbial invasion of the amniotic cavity (MIAC) and fetal infection occur.^4^, ^8^ Around 20–30% of women with spontaneous preterm birth exhibit MIAC despite intact fetal membranes, when sensitive PCR‐based detection tests are used.^8^ Gram‐negative bacteria associated with ascending infection and chorioamnionitis include *Escherichia coli* and *Fusobacterium* species. Other common bacteria associated with preterm delivery are the genital mycoplasmas, *Ureaplasma parvum* and *Mycoplasma hominis*, and Gram‐positive organisms such as *Streptococcus agalactiae*, *peptostreptococcus* spp. and *Gardnerella vaginalis* 18 that produce LTA or PGN.

Infection is associated with elevated accumulation of pro‐inflammatory cytokines in the amniotic fluid of preterm labouring women, with higher levels of IL‐1β, TNF and IL‐6 compared to women without infection.^11^ Increased fetal membrane expression of inflammatory signalling molecules, receptors and chemokines including CCL2, CCL3, CCL5, CCL20 and CXCL6 is implicated in regulating recruitment of inflammatory leucocytes and amplification of the inflammatory response.^10^, ^11^ Microbial products act via TLR4 ligation to stimulate inflammatory chemokine release from leucocytes in the amniotic fluid, with the consequence of recruiting more leucocytes from the maternal circulation to amplify the inflammatory pathways (Figure 2). TLR4 expression is upregulated by infection, in part due to infiltration of TLR4‐expressing leucocytes in tissues.^33^, ^34^

In preterm birth without infection, excessive DAMPs including HSP70, HMGB1, cell‐free DNA, uric acid and oxysterols released from stressed and dying gestational tissues are thought to provoke pro‐inflammatory activation.^40^, ^41^ These DAMPs accumulate faster after tissue injury than in on‐time labour, causing parturition to be prematurely triggered, particularly if anti‐inflammatory protective mechanisms are weak or insufficient.^4^, ^10^, ^18^ Many of these DAMPs are ligands for TLR4.^25^ Their accumulation is associated with, and may stimulate, elevated fetal lung synthesis of PAF and surfactant proteins.^42^, ^43^ that do not bind directly to TLR4, but require TLR4 for amplification of their pro‐inflammatory effects^44^, ^45^, ^46^, ^47^ (see later).

## Pamps induce TLR4‐mediated inflammation in preterm delivery

Mice with genetic deficiency in TLR4 provide compelling evidence that TLR4 is critical for preterm labour induced by Gram‐negative bacteria. C3H/HeJ mice that carry a spontaneous mutation in *Tlr4* do not deliver preterm after intrauterine administration of heat‐killed *E. coli*. In contrast, C3HeB/FeJ mice that express *Tlr4* exhibit 100% preterm delivery.^48^ Predictably, *Tlr4* ^−/−^ mice are also resistant to preterm labour induced by LPS.^23^ *Tlr4* ^−/−^ mice and C3H/HeJ mice both exhibit a lower fetal death rate and decreased placental necro‐inflammatory response after administration of *Fusobacterium nucleatum* compared to wild‐type mice.^49^ Interestingly, fetal inflammatory injury depends on fetal as well as maternal TLR4 signalling, since LPS in the maternal circulation accesses fetal tissues, and maternal *Tlr4* ^−/−^ null mutation does not protect wild‐type fetuses from inflammatory injury.^50^

In mice, MyD88 facilitates LPS‐induced preterm labour (in contrast to term labour), while TRIF appears dispensable at least for TLR4‐mediated triggers.^51^ *Myd88* ^−/−^ and *Myd88/Trif* ^−/−^ mice do not deliver preterm and exhibit normal fetal viability after administration of intrauterine heat‐killed *E. coli*.^51^ In contrast, *Trif* ^−/−^ and wild‐type mice are susceptible to preterm delivery and fetal death *in utero*.^51^ Nuclear NF‐κB is reduced in the uterus of *Myd88* ^−/−^ and *Myd88/Trif* ^−/−^ mice after *E. coli* delivery, accompanied by lower expression of *Il1b* and *Tnf* mRNA, compared to *Trif* ^−/−^ and wild‐type mice.^51^ There is evidence in mice that innate immune memory can alter sensitivity to TLR4 triggers. Activation of the type 1 IFN/IFNAR axis was shown to increase later susceptibility to LPS‐induced TLR4 activation, exacerbating pro‐inflammatory cytokine induction and reducing the dose of secondary inflammatory challenge required for preterm birth.^52^

### TLR4 signalling and inflammatory cytokines

Mouse models have been informative for defining the feed‐forward mechanisms that amplify TLR4 signals to promote uterine transformation and cause fetal injury. In late gestation pregnant mice, IL‐1α, IL‐1β, IL‐6 and TNF are synthesised in the uterus and fetal membranes after transcriptional activation as early as 3–4 h after LPS or *E. coli* administration.^53^, ^54^ These cytokines in turn drive upregulation of uterine activation genes *Ptgfr*, *Oxtr* and *Gja1* 53, 55 in a TLR4‐dependent manner.^23^, ^48^ In *Tlr4* ^−/−^ mice, expression of *Il1a*, *Il6*, *Il12b*, *Tnf* and *Il10* in fetal membranes, placenta and uterus is blunted after LPS administration, showing TLR4 is upstream and necessary for cytokine induction.^23^

IL‐1β signalling is critical, as demonstrated by the potent efficacy of IL‐1R antagonist to block preterm birth and fetal inflammatory injury after LPS exposure.^56^, ^57^ Additionally, mice with genetic deficiency in both the IL‐1β and TNF receptors (*Il1r1/Tnfrsf1a* ^−/−^) exhibit reduced preterm delivery in response to heat‐killed *E. coli*, compared to wild‐type mice.^58^ IL‐6 is also important, since anti‐IL‐6‐neutralising antibody and null mutation in the *Il6* gene both protect mice from LPS‐induced preterm birth.^55^, ^59^ IL‐6 and IL‐1β may amplify synergistic pathways since neither *Il6* nor *Il1b* null mutation fully abrogate susceptibility to preterm birth induced by intrauterine heat‐killed *E. coli*.^60^, ^61^

IL‐10 counteracts the effects of pro‐inflammatory cytokines to protect mice from preterm birth. Mice with *Il10* null mutation have greater susceptibility to LPS‐induced preterm birth.^53^, ^62^ IL‐10 suppresses pro‐inflammatory IFN‐γ and TNF production,^53^ and neutralising IFN‐γ and TNF protects *Il10* ^−/− ^mice from preterm delivery.^62^ In part, IL‐10 acts through stabilising an anti‐inflammatory phenotype in NK cells and T cells.^62^, ^63^

### TLR4 and inflammatory leucocytes

Mouse models also allow the biological roles of specific leucocyte lineages in preterm birth to be defined. After administration of a microbial stimulus, cytokine induction is rapidly followed by influx of inflammatory leucocytes into gestational tissues. This response resembles the immune cell changes that accompany term birth and are attributable to direct and indirect effects of TLR4 signalling. Innate immune cells are the first and largest component of the leucocyte infiltrate, with neutrophils arriving in the myometrium and decidua within hours after intraperitoneal LPS.^64^, ^65^ Intrauterine LPS administration causes a similar response, with accumulation in the fetal membrane, placenta and decidua before the myometrium.^66^ Surprisingly, depletion of neutrophils does not delay preterm delivery,^66^ implying that neutrophils are not essential for parturition.

Macrophages accumulate in the uterine decidua, but not myometrium, during LPS‐induced preterm birth.^64^, ^67^ They also contribute to tissue remodelling in the cervix, since macrophage depletion 4 h before intravaginal administration of LPS suppressed cervical collagen degradation and MMP9 activity, impeding progression to delivery.^68^

Cells of the adaptive immune response are also involved. LPS administration causes activation of T cells and NK cells in maternal blood and placenta in mice, while anti‐TLR4 antibody administration diminishes the T‐cell and NK cell changes.^69^ Mice deficient in invariant NK cells have an attenuated response to LPS‐induced preterm delivery, accompanied by a lower percentage of NK cells and T cells after LPS administration.^70^ This underscores a crucial role of invariant NK cells in activating decidual NK cells, dendritic cells and T cells involved in preterm birth.

A shift towards an immunogenic profile in T cells can reflect loss of immune tolerance, to increase susceptibility to preterm birth. Chronic chorioamnionitis characterised by excessive effector T cells is common in late preterm birth in women and is presumed to reflect excessive erosion of maternal allograft tolerance.^71^ T‐ and B‐cell‐deficient mice deficient in recombination activation gene (*Rag1* ^−/−^) are more susceptible to LPS‐induced preterm delivery than wild‐type mice.^72^ Deficiency in anti‐inflammatory Treg cells likely elevates susceptibility to LPS, since transfer of CD4^+^ T cells before LPS injection protected *Rag1* ^−/−^ mice from premature delivery.^72^ CD4^+^FOXP3^+^ uterine Treg cells normally decrease after LPS treatment,^67^ supporting the view that Treg cells suppress the inflammatory response and constrain premature delivery induced by TLR4 activation.

**Figure 4:**
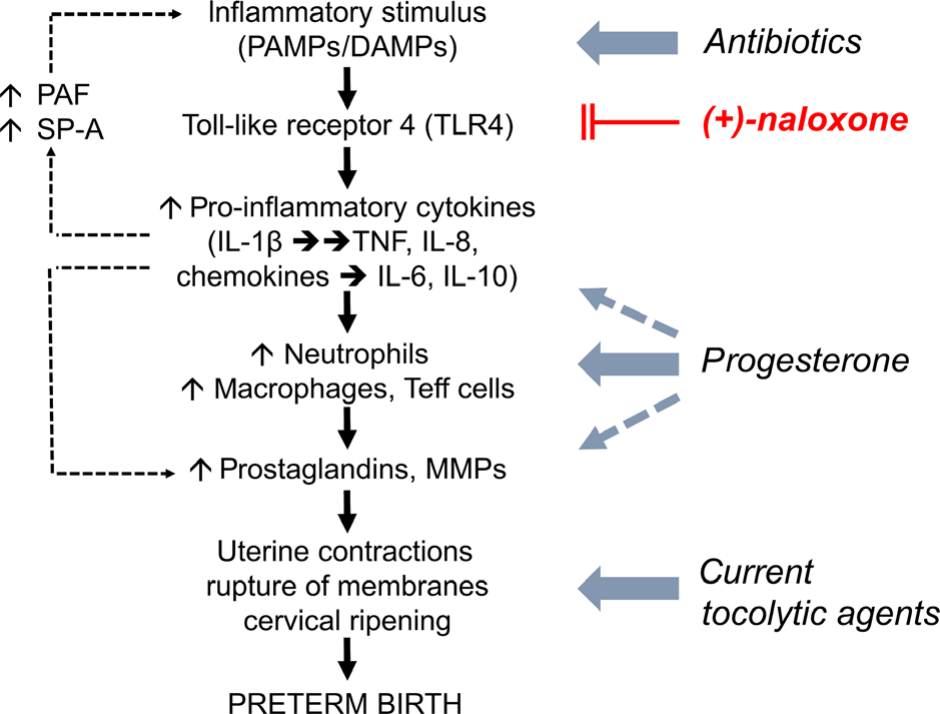
Toll‐like receptor‐4 (TLR4) signalling is an upstream driver of inflammation in spontaneous preterm labour. Exposure to pathogen‐associated molecular patterns (PAMPs) in the event of infection, or damage‐associated molecular patterns (DAMPs) in the event of sterile tissue insult or injury associated with oxidative stress, placental senescence or maternal immune imbalance, causes aberrant activation of TLR4 to initiate inflammation in preterm labour. Activated TLR4 acts to induce synthesis of pro‐inflammatory cytokines including IL‐1β, IL‐6, IL‐8 and TNF, which together with chemokines mediate recruitment of pro‐inflammatory leucocytes including neutrophils, macrophages and T cells. Platelet‐activating factor (PAF) and surfactant protein‐A (SP‐A), released from fetal lungs into amniotic fluid, further drive TLR4‐mediated cytokine induction to amplify the inflammatory cascade. In turn, inflammatory cells and mediators induce upregulation of uterine activation genes that cause uterine contractions, rupture of fetal membranes, and cervical ripening and dilation, to ultimately result in delivery of the fetus. Existing pharmacological strategies for delaying threatened preterm labour include antibiotics to limit microbial growth, progesterone to inhibit pro‐inflammatory mediators and anti‐tocolytic agents to suppress uterine contractions. (+)‐Naloxone compounds antagonise TLR4 activation, at the apex of the inflammatory cascade.

Together, the data from genetic mouse models provide evidence of a specific causal sequence in the roles of cytokines and immune cells leading to preterm birth. The results point to TLR4 signalling as a key effector at the apex of the inflammatory cascade and show TLR4 ligation causes MAPK and NF‐κB activation that induces IL‐1β and IL‐6 to elicit recruitment of pro‐inflammatory leucocytes, shift the phenotypes of regulatory immune cells and ultimately induce expression of uterine activation and cervical remodelling genes (Figure 4).

## Damps and TLR4 activation in preterm birth

Release of DAMPs from placental membranes occurs as a consequence of their progressive senescence in late gestation and is likely accelerated by fetal maturation signals.^40^ In the setting of preterm labour in the absence of infection, tissue damage causes inflammation and DAMP accumulation (Figure 2), after DAMP release from stressed or necrotic cells and their extracellular structures. In sites of infection, DAMPs accumulate in affected tissue and accelerate inflammation ensuing from PAMP‐induced TLR activation. Several DAMPs known to be ligands for TLR4 accumulate in gestational tissues prior to labour and become prematurely and more extensively increased in preterm labour. These endogenous TLR4 regulators likely signal through both MyD88 and TRIF‐dependent IRF1, and also via other pattern recognition receptors including receptor for advanced glycation end products (RAGE).^25^

### High‐mobility group box‐1 (HMGB1)

The chromatin‐associated protein HMGB1 is a well‐known DAMP released from stressed and necrotic cells. Both TLR4 and TLR2, as well as the RAGE receptor, can bind HMGB1 to trigger NF‐κB activation and inflammatory cytokine release.^73^ HMGB1 induces *TNF*, *IL6* and *PTGS2* expression in human myocytes,^74^ promotes *TLR2* and *TLR4* expression and amplifies IL‐1β, IL‐6 and TNF production, in human fetal membranes.^75^ HMGB1 concentrations are elevated in amniotic fluid of women at term in labour, especially in women with chorioamnionitis,^76^ due to activated macrophages that are an abundant source of HMGB1.^77^ Increased *HMGB1* transcription occurs in fetal membranes in preterm labour compared to normal term labour^75^ and is elevated by intra‐amniotic infection.^78^

Nonmicrobial insults such as oxidative stress, or premature fetal membrane ageing and senescence, also induce HMGB1.^74^, ^75^ Stretch is another trigger – studies using intra‐amniotic balloon inflation to mimic uterine overdistension to elicit preterm delivery in pigtail macaques show that stretch is associated with elevated amniotic fluid HMGB1 as well as cytokines, chemokines and prostaglandins.^79^

The fetus also contributes to HMGB1 production. HMGB1 is commonly detected after fetal injury induced by inflammation in humans and is a prominent DAMP at sites of fetal damage in mouse models.^80^ A key role for HMGB1 in premature parturition is indicated by experiments showing that preterm delivery and neonatal death are induced by HMGB1 administration to the amniotic cavity in mice.^81^

### Cell‐free DNA

Placental growth involves substantial release of microvesicle‐encapsulated, cell‐free fetal DNA‐containing apoptotic trophoblasts into the maternal circulation.^82^, ^83^ Substantial amounts of cell‐free DNA are shed as gestation progresses, reaching gram amounts per day. Particularly in late gestation, histone‐associated cell‐free DNA from the fetal membranes is released into amniotic fluid where it promotes inflammation and oxidative stress.^82^ Both the nucleic acid and protein (histone) constituents of the cell‐free DNA are pro‐inflammatory and comprise structures that are potent ligands of TLRs. TLR9 is implicated in mouse studies as a major mediator of fetal loss after inflammation induced by cell‐free DNA.^84^ However, in humans TLR9 exerts distinct functions and TLR4 and TLR2 are implicated as principal mediators of the response to cell‐free DNA.^85^

### Heat‐shock protein 70 (HSP70)

HSP70 is a well‐characterised HSP released from stressed and necrotic cells and is a known ligand for TLR4.^86^ The concentrations of HSP70 increase in amniotic fluid towards the end of gestation and during labour.^87^, ^88^ A positive association between serum HSP70 and gestational age is found in healthy pregnant women.^89^ HSP70 is detectable in the maternal decidua, as well as trophoblasts, Hofbauer and endothelial cells of the placenta.^90^ The extensive tissue remodelling and cell death prior to fetal membrane rupture are likely to promote release of HSP70 at term.^91^ Preterm labour is associated with elevated HSP70 concentrations in umbilical cord serum, placenta and maternal serum.^92^ In the event of infection, HSP70 release from human fetal membranes is further increased.^87^, ^93^ While HSP70 induces inflammatory cytokines through NF‐κB activation in other tissue systems,^86^ this has not been studied in gestational tissues. A recent study in mice showed intra‐amniotic HSP70 administration does not directly elicit preterm birth, but can induce fetal loss and adverse neonatal outcomes.^94^

### Uric acid

Uric acid has antioxidant activity at physiological levels, but when it accumulates can take on a crystalline particulate form that results in oxidative damage and activates inflammation via TLR4.^30^ Similar effects of elevated amniotic fluid uric acid originating in fetal urine occur in the placenta and gestational tissues, where uric acid induces IL‐1β in trophoblasts via inflammasome activation.^95^ A large cohort study has linked maternal hyperuricemia during the third trimester of pregnancy with preterm delivery, in women where infection and other clinical risk factors were absent.^96^

### Oxysterols

Oxysterols are pro‐inflammatory and pro‐apoptotic compounds formed when cholesterol oxidises in response to elevated levels of reactive oxygen species or increased activity of oxidative enzymes. Serum concentrations of oxidised low‐density lipoprotein carrying oxysterols are increased in pregnancy,^97^ particularly in preeclampsia^98^, ^99^ and fetal growth restriction.^100^ Two oxysterols, 25‐hydrocholesterol and 7‐ketocholesterol, act to impair trophoblast differentiation and fusion, and at high concentrations cause cell death.^101^ At non‐toxic concentrations, both 25‐hydrocholesterol and 7‐ketocholesterol elicit activation of placental TLR4 and induce IL‐6, CCL4 and TNF production in primary trophoblasts, in a TLR4‐dependent manner.^101^

## Fetal TLR4 regulators in preterm birth

In addition to DAMPs, other effector molecules released from fetal or placental tissues under sterile or infection‐associated conditions stimulate TLR4‐dependent pathways to promote parturition and preterm birth. These agents include PAF,^44^, ^45^ SP‐A and SP‐D,^47^ and fetal fibronectin and related extracellular matrix molecules.^102^ While these agents do not meet the classical definition of DAMPs as intracellular alarmins released upon cell stress or necrotic cell death, they nevertheless act to amplify pro‐inflammatory signalling in a TLR4‐dependent manner at parturition, as detailed below.

### Platelet‐activating factor (PAF)

A potent mediator of inflammation implicated in both sterile and infection‐associated preterm labour is the glycophospholipid factor PAF.^39^, ^45^ PAF is synthesised by alveolar type II cells in the fetal lung and accumulates in amniotic fluid prior to labour in mice^103^ and humans.^104^, ^105^ PAFR expression becomes progressively elevated in late gestation, in the uterus and cervix of mice,^106^ and the myometrium, cervix, placenta and fetal membrane of humans.^39^ Studies in mice show that fetal lung‐derived PAF in amniotic fluid contributes to uterine activation and transition to a contractile state,^107^ after elevating NF‐κB activation in uterine cells to elicit inflammatory cytokine synthesis.^39^, ^103^

Mice with genetic deficiency in PAF synthesis exhibit delayed labour.^103^ Intra‐amniotic administration of PAF reverts the phenotype to normal term parturition and induction of expression of contractile‐associated genes *Gja* and *Oxtr*.^103^ Intrauterine administration of carbamyl PAF (cPAF) in late gestation causes preterm delivery in CD‐1 mice.^106^

Similarly in women, PAF accumulates in amniotic fluid after release from the maturing fetal lung.^104^ PAF elicits upregulation of the uterine activation gene *PTGS2* in choriodecidual cells and stimulates contractile activity in myometrial cells. In the cervix, PAF induces secretion of pro‐inflammatory cytokines and MMP1.^39^ Amniotic fluid PAF is elevated in women with preterm delivery.^42^, ^108^

The feed‐forward effects of PAF on parturition depend on TLR4‐mediated inflammation. Mice with a genetic deficiency in TLR4 appear resistant to PAF‐induced preterm labour, with substantially reduced expression of IL‐6 and IL‐1β in decidual, myometrial and placental cells after cPAF administration.^109^ This likely reflects a requirement for TLR4 in amplifying inflammation induced by PAF, as several PAF‐induced mediators of inflammation are ligands for TLR4, or interacts with TLR4 signalling.^25^, ^30^ Immune cells are highly responsive to PAF‐induced TLR4 activation. Peritoneal macrophages from *Tlr4* ^−/−^ mice secrete less TNF and CCL5 after *in vitro* culture with cPAF, compared to WT controls,^44^ and in intestinal epithelial cells, cPAF activates TLR4 to drive robust pro‐inflammatory signalling.^45^

Platelet‐activating factor induces upregulation of TLR4 synthesis and enhances sensitivity to PAMP and DAMP ligation.^45^ An interaction between TLR4 and the PAF receptor is also implicated in amplifying responses induced by TLR2 signalling, such that Gram‐positive bacteria cause release of endogenous pro‐inflammatory mediators recognised by PAF receptor and TLR4.^28^ This explains how endogenous PAF acts to enhance infection‐induced inflammation in maternal and fetal tissues, to accelerate preterm delivery in mice.

Furthermore, PAF acts to amplify PAMP‐induced preterm birth. Elevated PAF secretion along with increased *Tnf*, *Il1b* and *Ccl5* expression is detected in the uterus, decidua and placenta of mice with a null mutation in PAF acetylhydrolase (*Paf/af* ^−/−^ mice), conferring greater susceptibility to preterm birth induced by heat‐killed *E. coli*.^44^ Administration of a PAF antagonist prior to intrauterine LPS also protects mice from preterm birth.^106^ Induction of preterm birth by cPAF requires TLR4 expression. *Tlr4* ^−/−^ mice have substantially lower preterm birth rates and a reduced placental and decidual cytokine response to cPAF.^109^

### Surfactant proteins

Surfactants are phospholipid‐rich proteins of the collectin family synthesised by pulmonary alveolar type II cells to reduce surface tension and enable mature lung function after birth.^103^ Both SP‐A and SP‐D exhibit capacity to modulate various aspects of the immune and inflammatory response, through mechanisms that at least partly depend on TLR4.^110^ Several studies indicate roles for SP‐A and SP‐D in regulating the timing of parturition and birth although the specific mechanisms are yet to be defined.^43^

Experiments in mice show that expression of *Spa* mRNA commences on gd 15 in the fetal lung and progressively increases until delivery 3–4 days later.^39^ SP‐A induces NF‐κB activation to elicit TNF and IL‐10 production^47^ and causes preterm birth when administered to the amniotic cavity in mice.^111^ Amniotic fluid macrophages are central mediators in this process – they respond to SP‐A by trafficking into the uterus, where they display activated nuclear NF‐κB and elevated IL‐1β.^111^ Remarkably, SP‐A and SP‐D deficiency in *Spa/d* ^−/−^ mice causes delayed parturition in the second pregnancy, but not the first pregnancy, implying an interaction with the immune response and/or tissue repair mechanisms affected by parity.^38^ *Spa/d* ^−/−^ mice have reduced myometrial expression of *Il1b* and *Il6* and the UAGs, *Gja* and *Oxtr*, in late gestation compared to wild‐type controls.^38^ In mice engineered to overexpress human SP‐A, elevated SP‐A protein in fetal lungs and amniotic fluid does not affect duration of pregnancy. However, LPS administration in late gestation elicited higher levels of TNF and IL‐10 in fetal and uterine tissues, suggesting that SP‐A acts to amplify intrauterine inflammatory mediators.^112^

The kinetics of SP‐A onset in the human fetal lung suggests a more complex role than in mice. SP‐A synthesis begins around the final 2 months of gestation, and amniotic fluid SP‐A accumulates progressively until term,^113^ but is lower in women in labour than women not in labour.^114^ In women with chorioamnionitis, SP‐A synthesis is elevated and appears to promote maturation of the fetal lung, reflected in lower fetal respiratory distress after birth.^114^ This may explain how chorioamnionitis promotes preterm labour, since SP‐A released into amniotic fluid has potential to target TLR on fetal membranes and stimulate production of PGE_2_.^115^ Myometrial cells also respond to SP‐A with increased PTGS2 synthesis,^116^ suggesting SP‐A sequestered into the myometrium might induce pro‐inflammatory cytokines and induce UAPs *in vivo*. In contrast, SP‐A exerts anti‐inflammatory effects in human amnion explants, acting to downregulate expression of *IL1B*, *CXCL2* and *CXCL5*.^117^ The different effector functions of SP‐A might be explained by different receptor protein interactions with microbial products.^43^ Concurrent administration of SP‐A and LPS decreased the preterm delivery rate compared to mice administered LPS alone,^118^ and SP‐A was associated with lower *Tnf*, *Il1b* and *Ccl5* expression in placenta and fetus. Further work is thus required to define how SP‐A interacts with TLR signalling induced by PAMPs to regulate inflammation in gestational tissues and the timing of labour.

## TLR4 as a target for preterm birth preventative therapeutics

Collectively, these studies provide convincing evidence that TLR4 is a key mediator of microbial and endogenous pro‐inflammatory effectors originating in the uterus, fetus and placenta, with a pivotal role in provoking parturition in a wide range of preterm and term scenarios. As well as infection‐associated preterm birth, TLR4 is implicated in sensing and amplifying amniotic fluid PAF and SP‐A, critical fetal signals that trigger parturition to coincide with fetal maturation and ability to survive *ex utero*,^39^ and a key receptor for HMGB1 and other DAMPs released by fetal membranes in response to senescence, injury and oxidative stress.^40^, ^41^ TLR4 is associated with leucocytes as well as non‐leucocytic cell lineages in the fetal membranes and so is ideally positioned to respond to the DAMPs and fetal signals in amniotic fluid. Leucocytes may be particularly sensitive – macrophages in the amniotic cavity express high levels of TLR4 and are known to amplify inflammation in late gestation, through release of pro‐inflammatory cytokines TNF and IL‐1β, and matrix‐remodelling enzymes that facilitate fetal membrane rupture during labour.

This scheme highlights TLR4 as an attractive drug target for delay or prevention of preterm birth (Figure 4). Studies in rodent and primate models provide evidence that blocking TLR4 signalling using bioactive or pharmaceutical agents is effective in preventing preterm delivery triggered by microbial or sterile stimuli. Blockade of TLR4 signalling with anti‐TLR4 monoclonal antibody reduces leucocyte activation and the incidence of preterm labour induced by LPS.^69^ Another TLR4 antagonist, lipid A mimetic CXR‐526, is effective in mice given *F. nucleatum*, a Gram‐negative bacterium that causes preterm birth and premature rupture of membranes in women.^49^ Although the lipid A mimetic did not inhibit bacterial colonisation of placental tissues, pro‐inflammatory cytokine expression and the extent of necrosis were reduced.^49^ Initial results in primate models are also promising. In rhesus monkeys, lipid A mimetic was effective in suppressing cytokines in amniotic fluid, as well as prostaglandin production and uterine contractile activity, without apparent side effects such as fever or complement activation.^119^

To date, most studies have concentrated on preterm birth as an endpoint rather than fetal outcomes. However, TLR4 antagonism may also be effective in prevention of fetal inflammatory injury resulting from preterm birth. Studies in rats show that after treatment to induce neonatal hyperoxia, a TLR4 antagonist LPS from the photosynthetic bacterium *Rhodobacter sphaeroides* (LPS‐RS) can prevent cardiac pro‐inflammatory cytokine induction and left ventricular hypertrophy and dysfunction.^120^

### (+)‐Naloxone and related compounds – novel TLR4 antagonists

Using mouse models, we have explored the utility of novel TLR4 antagonists of the (+)‐naloxone family as pharmacological interventions for preterm labour.^121^, ^122^ (+)‐Naloxone has anti‐inflammatory activity similar to that originally described for (−)‐naloxone, but unlike (−)‐naloxone, it does not bind opioid receptors and specifically antagonises TLR4 signalling.^121^ (+)‐Naloxone is a TLR4 antagonist, that is the positive isomer of the opioid receptor antagonist (−)‐naloxone,^121^ a well‐described non‐selective antagonist of the µ‐opioid receptor that is commonly prescribed for opioid addiction, including in pregnant women and neonates.^123^ (+)‐Naloxone does not have opioid actions, but binds MD‐2 to prevent TLR4 engaging with LPS or other ligands^124^ (Figure 3), thereby suppressing NF‐κB activation and IL‐1β, IL‐6 and TNF production.^125^, ^126^ In contrast to anti‐TLR4‐neutralising antibodies,^69^ (+)‐naloxone is a small molecule with potential to penetrate the placenta^127^ and has a pharmacokinetic profile suited to short systemic exposure or longer term delivery if required.

As a first approach, we tested the effect of (+)‐naloxone on birth outcomes in wild‐type mice. When (+)‐naloxone was given in late gestation, there were no adverse effects on pup health despite parturition and labour being delayed, consistent with an essential role for TLR4 in normal on‐time birth.^23^ We went on to demonstrate in an LPS model of preterm birth that (+)‐naloxone is highly effective in suppressing inflammatory cytokine induction and progression to preterm delivery, protecting against fetal death and postnatal loss.^128^

There are limitations of LPS models of preterm birth, given that clinically more than one TLR ligand would usually be involved. It is predictable that, as a TLR4 antagonist, (+)‐naloxone should be effective in blocking LPS actions. Therefore, it was important to evaluate the efficacy of (+)‐naloxone in other models. A similar protective effect of (+)‐naloxone was seen in preterm birth induced by intrauterine administration of heat‐killed *E. coli*, which more closely mimics the clinical situation. Furthermore, the local induction by *E. coli* of inflammatory cytokine genes *Il1b*, *Il6*, *Tnf* and *Il10* in fetal membranes was suppressed, and (+)‐naloxone similarly attenuated cytokine expression in the placenta, uterine myometrium and decidua.^128^ These data demonstrate that (+)‐naloxone is a highly effective inhibitor of the inflammatory cascade of preterm parturition in models of infection‐induced preterm birth. Importantly, pups born after (+)‐naloxone treatment were protected from antenatal and postnatal death, and exhibited survival rates to weaning and growth trajectories indistinguishable from control mice.^128^

We have also utilised (+)‐naltrexone, which is structurally and functionally similar to (+)‐naloxone,^121^, ^122^ to suppress cPAF‐induced preterm birth.^109^, ^129^ Using a dose of cPAF sufficient to cause preterm delivery in 65% of Balb/c mice, administration of (+)‐naltrexone at 12‐h intervals for 48 h following cPAF completely blocked preterm birth and maintained pup viability and birthweights. The elevated levels of IL‐1β, IL‐6 and IL‐10 otherwise seen in decidua and myometrium after cPAF treatment were suppressed by (+)‐naltrexone, consistent with a mechanism involving suppression of TLR4‐mediated inflammatory activation.

The high survival rates and lack of overt adverse impact of (+)‐naloxone treatment in pups are encouraging, although studies to investigate impact of (+)‐naloxone on fetal tissues susceptible to inflammatory injury are not yet completed. In a recent study, we showed that (+)‐naloxone can protect against adverse metabolic programming effects induced by fetal exposure to inflammatory mediators.^130^ In adult progeny born after LPS challenge *in utero*, male but not female offspring exhibited elevated adipose tissue mass, reduced muscle mass, and elevated plasma leptin concentrations at 20 weeks of age. These effects were largely reversed by co‐administration of (+)‐naloxone. LPS‐induced expression of *Il1a*, *Il1b*, *Il6*, *Tnf* and *Il10* in fetal brain, placental and uterine tissues, where (+)‐naloxone suppressed the LPS‐induced cytokine expression. Fetal sex‐specific regulation of cytokine expression was evident, with higher *Il1a*, *Il1b*, *Il6* and *Il10* induced by LPS in tissues associated with male fetuses and greater suppression by (+)‐naloxone of *Il6* in females. These data demonstrate that modulating TLR4 signalling with (+)‐naloxone protects against inflammatory diversion of fetal developmental programming *in utero*, associated with attenuation of gestational tissue cytokine expression in a fetal sex‐specific manner. Male fetuses often suffer more extensive damage or higher rates of fetal loss, with surviving fetuses experiencing a more debilitating legacy of inflammatory injury.^131^ The greater disposition of males to TLR4‐mediated inflammatory cytokine induction is consistent with a higher risk strategy than in females, where the same challenge induced a lower cytokine response that was more readily attenuated by TLR4 signalling inhibition. The results suggest that targeting TLR4 can be effective for protecting against developmental programming effects of fetal exposure to maternal inflammatory mediators.^130^ Future studies will focus not just on metabolism but also on neurocognitive and immune function, which like metabolic dysfunction are highly vulnerable to inflammation‐induced fetal programming.^132^

These data imply that (+)‐naloxone protects the developing fetus from cytokines synthesised locally in fetal tissues after LPS accesses the fetal circulation.^50^ Elevated inflammatory cytokines in maternal and placental tissues can also impact the fetus through indirect effects on placental vascular integrity, transport function and nutrient supply.^133^ It seems likely that (+)‐naloxone also protects the placenta from inflammatory damage, although this requires formal evaluation. Studies in the 1980s in women administered intrathecal morphine for labour pain relief indicate that although (−)‐naloxone crosses the placental barrier, there is no evident of teratogenicity or adverse fetal effects.^127^ It is not yet known whether the anti‐inflammatory protective effects of (+)‐naloxone are achieved by (+)‐naloxone acting directly in fetal tissues or by suppressing release of pro‐inflammatory DAMPs that would otherwise adversely affect the placenta or fetus.

(+)‐Naloxone, (+)‐naltrexone and related drugs may have clinical advantages compared to neutralising antibodies and other TLR4 antagonist compounds. (+)‐Naloxone potently blocks LPS‐induced TLR4‐mediated signalling in several non‐pregnancy models, suppressing NF‐κB activation and inhibiting TNF and IL‐1β induction in immune cells.^125^, ^134^ In humans, the closely related compound (−)‐naloxone has an established safety profile and is approved for use in pregnancy, with no known adverse neonatal effects.^135^, ^136^ Given the lack of opioid receptor activity of (+)‐naloxone, (+)‐naloxone has distinct pharmacodynamic advantages in a clinical setting over the currently available (−)‐naloxone. In particular, the stereoselectivity of opioid receptors^124^ would permit use of exogenous opioids for maternal pain relief in labour.

### Intervention strategies for TLR4 signalling inhibitors

These data indicate further studies are warranted to investigate small‐molecule inhibition of TLR‐driven inflammation as a strategy for fetal protection and delaying preterm birth. Three different intervention scenarios can be envisaged. Firstly, there is an urgent need for treatments to effectively curtail threatened preterm birth at clinical presentation, to prolong gestation and protect the fetus against inflammatory injury, while allowing a window of time for antibiotic therapy and corticosteroid treatment to promote fetal lung maturation. In the event of infection, TLR4 inhibitors could be administered together with antibiotics. Even during successful antibiotic therapy, substantial amounts of PAMPS are generated. A TLR4 inhibitor would reasonably be a useful adjunct to antibiotics, acting to suppress inflammatory activation.^137^

Secondly, TLR4 antagonists might have value as prophylactic agents that target the initiating triggers, and feed‐forward signals, to dampen or arrest parturition before overt symptoms arise. Predictive tests would be required to identify at‐risk women in early gestation, and allow tailored interventions appropriate to individual clinical parameters. TLR4 inhibitors may be particularly helpful in pregnant women at high risk of excessive inflammation as a result of exposures to environmental toxins or stressors, which can elevate TLR4 expression in the absence of infection.^138^, ^139^ Other at‐risk women can be identified on the basis of *TLR4* gene polymorphisms associated with an increased risk of spontaneous preterm delivery.^140^, ^141^

Thirdly, there is potential for TLR4 inhibitor use in preterm neonates to suppress progression of fetal inflammatory response syndrome.^20^ Sequalae of an *in utero* inflammatory insult include fetal and newborn brain white matter destruction, cerebral palsy, necrotising enterocolitis and chronic lung disease,^4^ causing neurodevelopmental disability and a range of recurrent health problems in childhood.^19^ Inflammation can provoke fetal brain injury even when inflammation is insufficient to activate parturition,^142^ indicating the risk of sustained exposure to inflammatory mediators *in utero*. These complications have lifelong consequences for the survivors of preterm birth, and treatments that safely and effectively reduce or resolve inflammation in the neonatal phase will deliver enormous benefit.^19^

## Conclusion and future directions

TLR4 agonists, either of microbial origin or originating endogenously after tissue injury, are clearly implicated in the pathophysiological mechanisms of spontaneous preterm birth, and fetal inflammatory response syndrome. Compelling preclinical studies show that TLR4 has a master role in parturition‐associated inflammation and is involved in its initiation, progression and persistence. TLR4 antagonism has a clear pharmacological advantage over current therapeutic strategies for treating preterm labour, in targeting the apex of the inflammatory cascade. Treatment scenarios including prophylactic and therapeutic administration to at‐risk women, or to premature neonates, can be envisaged. These alternative administration scenarios are associated with different clinical, pharmacological, and ethical challenges and imperatives.

A pharmacologic strategy targeting TLR4 would overcome an important limitation of existing tocolytic agents, such as prostaglandin inhibitors, that attempt to stem uterine maturation and contraction, or progesterone, which offers less effective anti‐inflammatory suppression and has limited efficacy^6^, ^10^ (Figure 4). These agents do not suppress the upstream origins of pro‐inflammatory activation and can alter homeostatic pathways necessary to maintain maternal and fetal organ function.^2^, ^143^ Stemming inflammation at the level of TLR4 activation is expected to provide an added benefit of protecting the fetus from inflammatory injury. Tocolytic agents that suppress uterine contractility, the final phase of labour, do not impact upstream pro‐inflammatory activity and so leave fetal tissues vulnerable to inflammatory cytokines.^2^, ^143^

Small‐molecule inhibitors of the (+)‐naloxone family are potential drug candidates that offer considerable promise and warrant further development. Amongst the benefits of this family of compounds is their relatively straightforward synthesis, stability during handling and transport, and potential suitability for use in low resource settings, where the majority of infant mortality occurs.^1^ Moreover, they readily penetrate gestational tissues and access the placenta and fetal compartments, and, on the basis of data from the negative isomer (−)‐naloxone, are predicted to be safe in pregnant women and in newborn infants.^135^, ^136^ Future studies are needed to investigate the safety and efficacy of (+)‐naloxone drugs, and appropriate formulations and administration protocols, in large animal models relevant to human, notably sheep and non‐human primates.^144^ In particular, the risk of non‐specific suppression of the immune response and its impact on maternal defence from infection will require evaluation.

A key consideration is the response of the neonate to *in utero* exposure to TLR4 inhibitors, and their protective effect in the fetus against the damaging actions of inflammatory mediators, and ongoing consequences of inflammatory injury after birth.^4^, ^19^ An important consideration is the physiological role of TLR4‐mediated pathways in normal fetal tissue maturation and any adverse impact of pharmacologic interference with this. Clearly, clinical progression of this work will require extensive investigation of the benefits and risks of pharmacological delay of preterm birth for infants, particularly effects on neurodevelopment, to evaluate the efficacy of interventions to reduce inflammatory injury *in utero*, and ensure the benefits outweigh the alternative strategy of delivery and neonatal intensive care. In this context, it is critical to appreciate the differences between acute treatment for women in suspected preterm labour and chronic treatment of women to prevent onset of inflammation‐associated complications, and to design studies accordingly.

## Conflict of interest

The authors declare no conflict of interest.

## References

[1] Rubens CE , Sadovsky Y , Muglia L , Gravett MG , Lackritz E , Gravett C . Prevention of preterm birth: harnessing science to address the global epidemic. Sci Transl Med 2014; 6: 262sr5. 25391484 10.1126/scitranslmed.3009871

[2] Iams JD . Prevention of preterm parturition. N Engl J Med 2014; 370: 1861. 10.1056/NEJMc1402822 24806178

[3] Challis JR , Lockwood CJ , Myatt L , Norman JE , Strauss JF 3rd , Petraglia F . Inflammation and pregnancy. Reprod Sci 2009; 16: 206–215. 19208789 10.1177/1933719108329095

[4] Romero R , Dey SK , Fisher SJ . Preterm labor: one syndrome, many causes. Science 2014; 345: 760–765. 25124429 10.1126/science.1251816 PMC4191866

[5] Keelan JA . Pharmacological inhibition of inflammatory pathways for the prevention of preterm birth. J Reprod Immunol 2011; 88: 176–184. 21236496 10.1016/j.jri.2010.11.003

[6] Elovitz MA . Anti‐inflammatory interventions in pregnancy: now and the future. Semin Fetal Neonatal Med 2006; 11: 327–332. 16828353 10.1016/j.siny.2006.03.005

[7] Ireland DJ , Nathan EA , Li S *et al* Preclinical evaluation of drugs to block inflammation‐driven preterm birth. Innate Immun 2017; 23: 20–33. 27821647 10.1177/1753425916672313

[8] Keelan JA . Intrauterine inflammatory activation, functional progesterone withdrawal, and the timing of term and preterm birth. J Reprod Immunol 2018; 125: 89–99. 29329080 10.1016/j.jri.2017.12.004

[9] Christiaens I , Zaragoza DB , Guilbert L , Robertson SA , Mitchell BF , Olson DM . Inflammatory processes in preterm and term parturition. J Reprod Immunol 2008; 79: 50–57. 18550178 10.1016/j.jri.2008.04.002

[10] Boyle AK , Rinaldi SF , Norman JE , Stock SJ . Preterm birth: Inflammation, fetal injury and treatment strategies. J Reprod Immunol 2017; 119: 62–66. 28122664 10.1016/j.jri.2016.11.008

[11] Keelan JA , Blumenstein M , Helliwell RJ , Sato TA , Marvin KW , Mitchell MD . Cytokines, prostaglandins and parturition–a review. Placenta 2003; 24(Suppl A): S33–S46. 12842412 10.1053/plac.2002.0948

[12] Schober L , Radnai D , Schmitt E , Mahnke K , Sohn C , Steinborn A . Term and preterm labor: decreased suppressive activity and changes in composition of the regulatory T‐cell pool. Immunol Cell Biol 2012; 90: 935–944. 22751216 10.1038/icb.2012.33

[13] Takeda J , Fang X , Olson DM . Pregnant human peripheral leukocyte migration during several late pregnancy clinical conditions: a cross‐sectional observational study. BMC Pregnancy Childbirth 2017; 17: 16. 28068953 10.1186/s12884-016-1204-5 PMC5223432

[14] Heng YJ , Pennell CE , Chua HN , Perkins JE , Lye SJ . Whole blood gene expression profile associated with spontaneous preterm birth in women with threatened preterm labor. PLoS One 2014; 9: e96901. 24828675 10.1371/journal.pone.0096901 PMC4020779

[15] Yuan M , Jordan F , McInnes IB , Harnett MM , Norman JE . Leukocytes are primed in peripheral blood for activation during term and preterm labour. Mol Hum Reprod 2009; 15: 713–724. 19628509 10.1093/molehr/gap054 PMC2762373

[16] Okabe H , Makino S , Kato K , Matsuoka K , Seki H , Takeda S . The effect of progesterone on genes involved in preterm labor. J Reprod Immunol 2014; 104–105: 80–91. 10.1016/j.jri.2014.03.008 24933116

[17] Marcellin L , Schmitz T , Messaoudene M *et al* Immune modifications in fetal membranes overlying the cervix precede parturition in humans. J Immunol 2017; 198: 1345–1356. 28031337 10.4049/jimmunol.1601482

[18] Romero R , Espinoza J , Goncalves LF , Kusanovic JP , Friel L , Hassan S . The role of inflammation and infection in preterm birth. Semin Reprod Med 2007; 25: 21–39. 17205421 10.1055/s-2006-956773 PMC8324073

[19] Saigal S , Doyle LW . An overview of mortality and sequelae of preterm birth from infancy to adulthood. Lancet 2008; 371: 261–269. 18207020 10.1016/S0140-6736(08)60136-1

[20] Gotsch F , Romero R , Kusanovic JP *et al* The fetal inflammatory response syndrome. Clin Obstet Gynecol 2007; 50: 652–683. 17762416 10.1097/GRF.0b013e31811ebef6

[21] Koga K , Mor G . Expression and function of toll‐like receptors at the maternal‐fetal interface. Reprod Sci 2008; 15: 231–242. 18421019 10.1177/1933719108316391

[22] Abrahams VM , Mor G . Toll‐like receptors and their role in the trophoblast. Placenta 2005; 26: 540–547. 15993703 10.1016/j.placenta.2004.08.010

[23] Wahid HH , Dorian C , Chin PY *et al* Toll‐like receptor 4 is an essential upstream regulator of on‐time parturition and perinatal viability in mice. Endocrinology 2015; 156: 3828–3841. 26151355 10.1210/en.2015-1089 PMC4588813

[24] Koga K , Izumi G , Mor G , Fujii T , Osuga Y . Toll‐like receptors at the maternal‐fetal interface in normal pregnancy and pregnancy complications. Am J Reprod Immunol 2014; 72: 192–205. 24754320 10.1111/aji.12258

[25] Piccinini AM , Midwood KS . DAMPening inflammation by modulating TLR signalling. Mediators Inflamm 2010; 2010: 1–21. 10.1155/2010/672395 PMC2913853 20706656

[26] Strauss JF 3rd , Romero R , Gomez‐Lopez N *et al* Spontaneous preterm birth: advances toward the discovery of genetic predisposition. Am J Obstet Gynecol 2018; 218: 294–314.e2. 29248470 10.1016/j.ajog.2017.12.009 PMC5834399

[27] Dowling JK , Mansell A . Toll‐like receptors: the swiss army knife of immunity and vaccine development. Clin Transl Immunology 2016; 5: e85. 27350884 10.1038/cti.2016.22 PMC4910119

[28] Knapp S , von Aulock S , Leendertse M *et al* Lipoteichoic acid‐induced lung inflammation depends on TLR2 and the concerted action of TLR4 and the platelet‐activating factor receptor. J Immunol 2008; 180: 3478–3484. 18292574 10.4049/jimmunol.180.5.3478

[29] Nahid MA , Satoh M , Chan EK . MicroRNA in TLR signaling and endotoxin tolerance. Cell Mol Immunol 2011; 8: 388–403. 21822296 10.1038/cmi.2011.26 PMC3618661

[30] Rock KL , Latz E , Ontiveros F , Kono H . The sterile inflammatory response. Annu Rev Immunol 2010; 28: 321–342. 20307211 10.1146/annurev-immunol-030409-101311 PMC4315152

[31] Kim YM , Romero R , Chaiworapongsa T *et al* Toll‐like receptor‐2 and ‐4 in the chorioamniotic membranes in spontaneous labor at term and in preterm parturition that are associated with chorioamnionitis. Am J Obstet Gynecol 2004; 191: 1346–1355. 15507964 10.1016/j.ajog.2004.07.009

[32] Fazeli A , Bruce C , Anumba DO . Characterization of Toll‐like receptors in the female reproductive tract in humans. Hum Reprod 2005; 20: 1372–1378. 15695310 10.1093/humrep/deh775

[33] Eriksson M , Meadows SK , Basu S , Mselle TF , Wira CR , Sentman CL . TLRs mediate IFN‐γ production by human uterine NK cells in endometrium. J Immunol 2006; 176: 6219–6224. 16670332 10.4049/jimmunol.176.10.6219

[34] Kumazaki K , Nakayama M , Yanagihara I , Suehara N , Wada Y . Immunohistochemical distribution of Toll‐like receptor 4 in term and preterm human placentas from normal and complicated pregnancy including chorioamnionitis. Hum Pathol 2004; 35: 47–54. 14745724 10.1016/j.humpath.2003.08.027

[35] Gonzalez JM , Xu H , Ofori E , Elovitz MA . Toll‐like receptors in the uterus, cervix, and placenta: is pregnancy an immunosuppressed state? Am J Obstet Gynecol 2007; 197: 296.e1–296.e6. 17826427 10.1016/j.ajog.2007.06.021

[36] Harju K , Ojaniemi M , Rounioja S *et al* Expression of toll‐like receptor 4 and endotoxin responsiveness in mice during perinatal period. Pediatr Res 2005; 57: 644–648. 15718365 10.1203/01.PDR.0000156212.03459.A9

[37] Yamamoto M , Sato S , Hemmi H *et al* Role of adaptor TRIF in the MyD88‐independent toll‐like receptor signaling pathway. Science 2003; 301: 640–643. 12855817 10.1126/science.1087262

[38] Montalbano AP , Hawgood S , Mendelson CR . Mice deficient in surfactant protein A (SP‐A) and SP‐D or in TLR2 manifest delayed parturition and decreased expression of inflammatory and contractile genes. Endocrinology 2013; 154: 483–498. 23183169 10.1210/en.2012-1797 PMC3529364

[39] Mendelson CR , Montalbano AP , Gao L . Fetal‐to‐maternal signaling in the timing of birth. J Steroid Biochem Mol Biol 2017; 170: 19–27. 27629593 10.1016/j.jsbmb.2016.09.006 PMC5346347

[40] Menon R , Bonney EA , Condon J , Mesiano S , Taylor RN . Novel concepts on pregnancy clocks and alarms: redundancy and synergy in human parturition. Hum Reprod Update 2016; 22: 535–560. 27363410 10.1093/humupd/dmw022 PMC5001499

[41] Nadeau‐Vallee M , Obari D , Palacios J *et al* Sterile inflammation and pregnancy complications: a review. Reproduction 2016; 152: R277–R292. 27679863 10.1530/REP-16-0453

[42] Hoffman DR , Romero R , Johnston JM . Detection of platelet‐activating factor in amniotic fluid of complicated pregnancies. Am J Obstet Gynecol 1990; 162: 525–528. 2309839 10.1016/0002-9378(90)90423-5

[43] Yadav AK , Madan T , Bernal AL . Surfactant proteins A and D in pregnancy and parturition. Front Biosci (Elite Ed) 2011; 3: 291–300. 21196309 10.2741/e244

[44] Agrawal V , Jaiswal MK , Ilievski V , Beaman KD , Jilling T , Hirsch E . Platelet‐activating factor: a role in preterm delivery and an essential interaction with Toll‐like receptor signaling in mice. Biol Reprod 2014; 91: 119. 25253732 10.1095/biolreprod.113.116012 PMC4434927

[45] Soliman A , Michelsen KS , Karahashi H *et al* Platelet‐activating factor induces TLR4 expression in intestinal epithelial cells: implication for the pathogenesis of necrotizing enterocolitis. PLoS One 2010; 5: e15044. 20976181 10.1371/journal.pone.0015044 PMC2955554

[46] Ohya M , Nishitani C , Sano H *et al* Human pulmonary surfactant protein D binds the extracellular domains of Toll‐like receptors 2 and 4 through the carbohydrate recognition domain by a mechanism different from its binding to phosphatidylinositol and lipopolysaccharide. Biochemistry 2006; 45: 8657–8664. 16834340 10.1021/bi060176z

[47] Guillot L , Balloy V , McCormack FX , Golenbock DT , Chignard M , Si‐Tahar M . Cutting edge: the immunostimulatory activity of the lung surfactant protein‐A involves Toll‐like receptor 4. J Immunol 2002; 168: 5989–5992. 12055204 10.4049/jimmunol.168.12.5989

[48] Wang H , Hirsch E . Bacterially‐induced preterm labor and regulation of prostaglandin‐metabolizing enzyme expression in mice: the role of toll‐like receptor 4. Biol Reprod 2003; 69: 1957–1963. 12904319 10.1095/biolreprod.103.019620

[49] Liu H , Redline RW , Han YW . Fusobacterium nucleatum induces fetal death in mice via stimulation of TLR4‐mediated placental inflammatory response. J Immunol 2007; 179: 2501–2508. 17675512 10.4049/jimmunol.179.4.2501

[50] Brown AG , Maubert ME , Anton L , Heiser LM , Elovitz MA . The tracking of lipopolysaccharide through the feto‐maternal compartment and the involvement of maternal TLR4 in inflammation‐induced fetal brain injury. Am J Reprod Immunol 2019; 82: e13189. 31495009 10.1111/aji.13189 PMC6899932

[51] Filipovich Y , Lu SJ , Akira S , Hirsch E . The adaptor protein MyD88 is essential for *E coli*‐induced preterm delivery in mice. Am J Obstet Gynecol 2009; 200: 93.e1–93.e8. 19121660 10.1016/j.ajog.2008.08.038

[52] Cappelletti M , Presicce P , Lawson MJ *et al* Type I interferons regulate susceptibility to inflammation‐induced preterm birth. JCI Insight 2017; 2: e91288. 28289719 10.1172/jci.insight.91288 PMC5333966

[53] Robertson SA , Skinner RJ , Care AS . Essential role for IL‐10 in resistance to lipopolysaccharide‐induced preterm labor in mice. J Immunol 2006; 177: 4888–4896. 16982931 10.4049/jimmunol.177.7.4888

[54] Hirsch E , Saotome I , Hirsh D . A model of intrauterine infection and preterm delivery in mice. Am J Obstet Gynecol 1995; 172: 1598–1603. 7538729 10.1016/0002-9378(95)90503-0

[55] Robertson SA , Christiaens I , Dorian CL *et al* Interleukin‐6 is an essential determinant of on‐time parturition in the mouse. Endocrinology 2010; 151: 3996–4006. 20610570 10.1210/en.2010-0063

[56] Nadeau‐Vallee M , Quiniou C , Palacios J *et al* Novel noncompetitive IL‐1 receptor‐biased ligand prevents infection‐ and inflammation‐induced preterm birth. J Immunol 2015; 195: 3402–3415. 26304990 10.4049/jimmunol.1500758

[57] Nadeau‐Vallee M , Chin PY , Belarbi L *et al* Antenatal suppression of IL‐1 protects against inflammation‐induced fetal injury and improves neonatal and developmental outcomes in mice. J Immunol 2017; 198: 2047–2062. 28148737 10.4049/jimmunol.1601600

[58] Hirsch E , Filipovich Y , Mahendroo M . Signaling via the type I IL‐1 and TNF receptors is necessary for bacterially induced preterm labor in a murine model. Am J Obstet Gynecol 2006; 194: 1334–1340. 16647919 10.1016/j.ajog.2005.11.004

[59] Wakabayashi A , Sawada K , Nakayama M *et al* Targeting interleukin‐6 receptor inhibits preterm delivery induced by inflammation. Mol Hum Reprod 2013; 19: 718–726. 23969038 10.1093/molehr/gat057

[60] Reznikov LL , Fantuzzi G , Selzman CH *et al* Utilization of endoscopic inoculation in a mouse model of intrauterine infection‐induced preterm birth: role of interleukin 1β. Biol Reprod 1999; 60: 1231–1238. 10208989 10.1095/biolreprod60.5.1231

[61] Yoshimura K , Hirsch E . Interleukin‐6 is neither necessary nor sufficient for preterm labor in a murine infection model. J Soc Gynecol Investig 2003; 10: 423–427. 10.1016/s1071-5576(03)00138-2 14519484

[62] Murphy SP , Hanna NN , Fast LD *et al* Evidence for participation of uterine natural killer cells in the mechanisms responsible for spontaneous preterm labor and delivery. Am J Obstet Gynecol 2009; 200: 308.e1–308.e9. 19114277 10.1016/j.ajog.2008.10.043 PMC3893044

[63] Prins JR , Zhang B , Schjenken JE , Guerin LR , Barry SC , Robertson SA . Unstable Foxp3+ regulatory T cells and altered dendritic cells are associated with lipopolysaccharide‐induced fetal loss in pregnant interleukin 10‐deficient mice. Biol Reprod 2015; 93: 95. 26224007 10.1095/biolreprod.115.128694

[64] Shynlova O , Nedd‐Roderique T , Li Y , Dorogin A , Nguyen T , Lye SJ . Infiltration of myeloid cells into decidua is a critical early event in the labour cascade and post‐partum uterine remodelling. J Cell Mol Med 2013; 17: 311–324. 23379349 10.1111/jcmm.12012 PMC3822594

[65] Shynlova O , Nedd‐Roderique T , Li Y , Dorogin A , Lye SJ . Myometrial immune cells contribute to term parturition, preterm labour and post‐partum involution in mice. J Cell Mol Med 2013; 17: 90–102. 23205502 10.1111/j.1582-4934.2012.01650.x PMC3823139

[66] Rinaldi SF , Catalano RD , Wade J , Rossi AG , Norman JE . Decidual neutrophil infiltration is not required for preterm birth in a mouse model of infection‐induced preterm labor. J Immunol 2014; 192: 2315–2325. 24501200 10.4049/jimmunol.1302891 PMC3932811

[67] Arenas‐Hernandez M , Romero R , St Louis D , Hassan SS , Kaye EB , Gomez‐Lopez N . An imbalance between innate and adaptive immune cells at the maternal‐fetal interface occurs prior to endotoxin‐induced preterm birth. Cell Mol Immunol 2015; 13: 462–473. 25849119 10.1038/cmi.2015.22 PMC4947814

[68] Gonzalez JM , Franzke CW , Yang F , Romero R , Girardi G . Complement activation triggers metalloproteinases release inducing cervical remodeling and preterm birth in mice. Am J Pathol 2011; 179: 838–849. 21801872 10.1016/j.ajpath.2011.04.024 PMC3157168

[69] Li L , Kang J , Lei W . Role of Toll‐like receptor 4 in inflammation‐induced preterm delivery. Mol Hum Reprod 2010; 16: 267–272. 19995880 10.1093/molehr/gap106

[70] Li LP , Fang YC , Dong GF , Lin Y , Saito S . Depletion of invariant NKT cells reduces inflammation‐induced preterm delivery in mice. J Immunol 2012; 188: 4681–4689. 22467647 10.4049/jimmunol.1102628

[71] Kim CJ , Romero R , Chaemsaithong P , Kim JS . Chronic inflammation of the placenta: definition, classification, pathogenesis, and clinical significance. Am J Obstet Gynecol 2015; 213: S53–S69. 26428503 10.1016/j.ajog.2015.08.041 PMC4782598

[72] Bizargity P , Del Rio R , Phillippe M , Teuscher C , Bonney EA . Resistance to lipopolysaccharide‐induced preterm delivery mediated by regulatory T cell function in mice. Biol Reprod 2009; 80: 874–881. 19144956 10.1095/biolreprod.108.074294 PMC2804837

[73] Klune JR , Dhupar R , Cardinal J , Billiar TR , Tsung A . HMGB1: endogenous danger signaling. Mol Med 2008; 14: 476–484. 18431461 10.2119/2008-00034.Klune PMC2323334

[74] Menon R , Behnia F , Polettini J , Saade GR , Campisi J , Velarde M . Placental membrane aging and HMGB1 signaling associated with human parturition. Aging (Albany NY) 2016; 8: 216–230. 26851389 10.18632/aging.100891 PMC4789578

[75] Bredeson S , Papaconstantinou J , Deford JH *et al* HMGB1 promotes a p38MAPK associated non‐infectious inflammatory response pathway in human fetal membranes. PLoS One 2014; 9: e113799. 25469638 10.1371/journal.pone.0113799 PMC4254744

[76] Romero R , Chaiworapongsa T , Savasan ZA *et al* Clinical chorioamnionitis is characterized by changes in the expression of the alarmin HMGB1 and one of its receptors, sRAGE. J Matern Fetal Neonatal Med 2012; 25: 558–567. 22578261 10.3109/14767058.2011.599083 PMC3914307

[77] Chen G , Li J , Ochani M *et al* Bacterial endotoxin stimulates macrophages to release HMGB1 partly through CD14‐ and TNF‐dependent mechanisms. J Leukoc Biol 2004; 76: 994–1001. 15331624 10.1189/jlb.0404242

[78] Romero R , Chaiworapongsa T , Alpay Savasan Z *et al* Damage‐associated molecular patterns (DAMPs) in preterm labor with intact membranes and preterm PROM: a study of the alarmin HMGB1. J Matern Fetal Neonatal Med 2011; 24: 1444–1455. 21958433 10.3109/14767058.2011.591460 PMC3419589

[79] Adams Waldorf KM , Singh N , Mohan AR *et al* Uterine overdistention induces preterm labor mediated by inflammation: observations in pregnant women and nonhuman primates. Am J Obstet Gynecol 2015; 213: 830.e1–830.e19. 26284599 10.1016/j.ajog.2015.08.028 PMC4679421

[80] Buhimschi CS , Baumbusch MA , Dulay AT *et al* Characterization of RAGE, HMGB1, and S100β in inflammation‐induced preterm birth and fetal tissue injury. Am J Pathol 2009; 175: 958–975. 19679874 10.2353/ajpath.2009.090156 PMC2731116

[81] Gomez‐Lopez N , Romero R , Plazyo O *et al* Intra‐amniotic administration of HMGB1 induces spontaneous preterm labor and birth. Am J Reprod Immunol 2016; 75: 3–7. 26781934 10.1111/aji.12443 PMC5029853

[82] Huppertz B , Kingdom JC . Apoptosis in the trophoblast–role of apoptosis in placental morphogenesis. J Soc Gynecol Investig 2004; 11: 353–362. 10.1016/j.jsgi.2004.06.002 15350247

[83] Taglauer ES , Wilkins‐Haug L , Bianchi DW . Review: cell‐free fetal DNA in the maternal circulation as an indication of placental health and disease. Placenta 2014; 35(Suppl): S64–S68. 24388429 10.1016/j.placenta.2013.11.014 PMC4886648

[84] Scharfe‐Nugent A , Corr SC , Carpenter SB *et al* TLR9 provokes inflammation in response to fetal DNA: mechanism for fetal loss in preterm birth and preeclampsia. J Immunol 2012; 188: 5706–5712. 22544937 10.4049/jimmunol.1103454

[85] Marsman G , Zeerleder S , Luken BM . Extracellular histones, cell‐free DNA, or nucleosomes: differences in immunostimulation. Cell Death Dis 2016; 7: e2518. 27929534 10.1038/cddis.2016.410 PMC5261016

[86] Asea A , Rehli M , Kabingu E *et al* Novel signal transduction pathway utilized by extracellular HSP70: role of toll‐like receptor (TLR) 2 and TLR4. J Biol Chem 2002; 277: 15028–15034. 11836257 10.1074/jbc.M200497200

[87] Chaiworapongsa T , Erez O , Kusanovic JP *et al* Amniotic fluid heat shock protein 70 concentration in histologic chorioamnionitis, term and preterm parturition. J Matern Fetal Neonatal Med 2008; 21: 449–461. 18570125 10.1080/14767050802054550 PMC2517420

[88] Jean‐Pierre C , Perni SC , Bongiovanni AM *et al* Extracellular 70‐kd heat shock protein in mid‐trimester amniotic fluid and its effect on cytokine production by *ex vivo*‐cultured amniotic fluid cells. Am J Obstet Gynecol 2006; 194: 694–698. 16522399 10.1016/j.ajog.2006.01.066

[89] Molvarec A , Rigo J Jr , Nagy B *et al* Serum heat shock protein 70 levels are decreased in normal human pregnancy. J Reprod Immunol 2007; 74: 163–169. 17296233 10.1016/j.jri.2006.12.002

[90] Shah M , Stanek J , Handwerger S . Differential localization of heat shock proteins 90, 70, 60 and 27 in human decidua and placenta during pregnancy. Histochem J 1998; 30: 509–518. 10192534 10.1023/a:1003259907014

[91] Kim S , Kwon J . Thymosin β4 has a major role in dermal burn wound healing that involves actin cytoskeletal remodelling via heat‐shock protein 70. J Tissue Eng Regen Med 2015; 11: 1262–1273. 25921810 10.1002/term.2028

[92] Chang A , Zhang Z , Jia L , Zhang L , Gao Y , Zhang L . Alteration of heat shock protein 70 expression levels in term and preterm delivery. J Matern Fetal Neonatal Med 2013; 26: 1581–1585. 23581516 10.3109/14767058.2013.795535

[93] Romero R , Miranda J , Kusanovic JP *et al* Clinical chorioamnionitis at term I: microbiology of the amniotic cavity using cultivation and molecular techniques. J Perinat Med 2015; 43: 19–36. 25720095 10.1515/jpm-2014-0249 PMC5881909

[94] Schwenkel G , Romero R , Slutsky R , Motomura K , Hsu CD , Gomez‐Lopez N . HSP70: an alarmin that does not induce high rates of preterm birth but does cause adverse neonatal outcomes. J Matern Fetal Neonatal Med 2020; 70: 1–9. 10.1080/14767058.2019.1706470 PMC7768925 31906756

[95] Mulla MJ , Myrtolli K , Potter J *et al* Uric acid induces trophoblast IL‐1β production via the inflammasome: implications for the pathogenesis of preeclampsia. Am J Reprod Immunol 2011; 65: 542–548. 21352397 10.1111/j.1600-0897.2010.00960.x PMC3114103

[96] Amini E , Sheikh M , Hantoushzadeh S , Shariat M , Abdollahi A , Kashanian M . Maternal hyperuricemia in normotensive singleton pregnancy, a prenatal finding with continuous perinatal and postnatal effects, a prospective cohort study. BMC Pregnancy Childbirth 2014; 14: 104. 24636149 10.1186/1471-2393-14-104 PMC3995428

[97] Makedou K , Kourtis A , Gkiomisi A *et al* Oxidized low‐density lipoprotein and adiponectin levels in pregnancy. Gynecol Endocrinol 2011; 27: 1070–1073. 21504339 10.3109/09513590.2011.569793

[98] Uzun H , Benian A , Madazli R , Topcuoglu MA , Aydin S , Albayrak M . Circulating oxidized low‐density lipoprotein and paraoxonase activity in preeclampsia. Gynecol Obstet Invest 2005; 60: 195–200. 16088195 10.1159/000087205

[99] Kim YJ , Park H , Lee HY *et al* Paraoxonase gene polymorphism, serum lipid, and oxidized low‐density lipoprotein in preeclampsia. Eur J Obstet Gynecol Reprod Biol 2007; 133: 47–52. 16949193 10.1016/j.ejogrb.2006.07.046

[100] Leduc L , Delvin E , Ouellet A *et al* Oxidized low‐density lipoproteins in cord blood from neonates with intra‐uterine growth restriction. Eur J Obstet Gynecol Reprod Biol 2011; 156: 46–49. 21324580 10.1016/j.ejogrb.2011.01.007

[101] Aye IL , Waddell BJ , Mark PJ , Keelan JA . Oxysterols exert proinflammatory effects in placental trophoblasts via TLR4‐dependent, cholesterol‐sensitive activation of NF‐κB. Mol Hum Reprod 2012; 18: 341–353. 22238372 10.1093/molehr/gas001

[102] Mogami H , Kishore AH , Shi H , Keller PW , Akgul Y , Word RA . Fetal fibronectin signaling induces matrix metalloproteases and cyclooxygenase‐2 (COX‐2) in amnion cells and preterm birth in mice. J Biol Chem 2013; 288: 1953–1966. 23184961 10.1074/jbc.M112.424366 PMC3548503

[103] Gao L , Rabbitt EH , Condon JC *et al* Steroid receptor coactivators 1 and 2 mediate fetal‐to‐maternal signaling that initiates parturition. J Clin Invest 2015; 125: 2808–2824. 26098214 10.1172/JCI78544 PMC4563678

[104] Billah MM , Johnston JM . Identification of phospholipid platelet‐activating factor (1‐0‐alkyl‐2‐acetyl‐sn‐glycero‐3‐phosphocholine) in human amniotic fluid and urine. Biochem Biophys Res Commun 1983; 113: 51–58. 6407483 10.1016/0006-291x(83)90430-8

[105] Hoffman DR , Truong CT , Johnston JM . The role of platelet‐activating factor in human fetal lung maturation. Am J Obstet Gynecol 1986; 155: 70–75. 3728606 10.1016/0002-9378(86)90081-5

[106] Elovitz MA , Wang Z , Chien EK , Rychlik DF , Phillippe M . A new model for inflammation‐induced preterm birth: the role of platelet‐activating factor and Toll‐like receptor‐4. Am J Pathol 2003; 163: 2103–2111. 14578208 10.1016/S0002-9440(10)63567-5 PMC1892431

[107] Kim BK , Ozaki H , Lee SM , Karaki H . Increased sensitivity of rat myometrium to the contractile effect of platelet activating factor before delivery. Br J Pharmacol 1995; 115: 1211–1214. 7582547 10.1111/j.1476-5381.1995.tb15027.x PMC1908802

[108] Silver RK , Caplan MS , Kelly AM . Amniotic fluid platelet‐activating factor (PAF) is elevated in patients with tocolytic failure and preterm delivery. Prostaglandins 1992; 43: 181–187. 1542743 10.1016/0090-6980(92)90085-8

[109] Wahid HH , Chin PY , Sharkey DJ , *et al* Toll‐like receptor‐4 antagonist (+)‐naltrexone protects against carbamyl‐platelet activating factor (cPAF)‐induced preterm labor in mice. Am J Pathol 2020; e‐pub ahead of print; 10.1016/j.ajpath.2020.01.008 PMC7237831 32084361

[110] Hartshorn KL . Role of surfactant protein A and D (SP‐A and SP‐D) in human antiviral host defense. Front Biosci (Schol Ed) 2010; 2: 527–546. 20036966 10.2741/s83

[111] Condon JC , Jeyasuria P , Faust JM , Mendelson CR . Surfactant protein secreted by the maturing mouse fetal lung acts as a hormone that signals the initiation of parturition. Proc Natl Acad Sci USA 2004; 101: 4978–4983. 15044702 10.1073/pnas.0401124101 PMC387359

[112] Salminen A , Vuolteenaho R , Paananen R , Ojaniemi M , Hallman M . Surfactant protein A modulates the lipopolysaccharide‐induced inflammatory response related to preterm birth. Cytokine 2011; 56: 442–449. 21865055 10.1016/j.cyto.2011.07.025

[113] Mendelson CR , Boggaram V . Hormonal and developmental regulation of pulmonary surfactant synthesis in fetal lung. Baillieres Clin Endocrinol Metab 1990; 4: 351–378. 2248600 10.1016/s0950-351x(05)80055-2

[114] Chaiworapongsa T , Hong JS , Hull WM *et al* The concentration of surfactant protein‐A in amniotic fluid decreases in spontaneous human parturition at term. J Matern Fetal Neonatal Med 2008; 21: 652–659. 18828058 10.1080/14767050802215193 PMC3418916

[115] Lopez Bernal A , Newman GE , Phizackerley PJ , Turnbull AC . Surfactant stimulates prostaglandin E production in human amnion. Br J Obstet Gynaecol 1988; 95: 1013–1017. 3191038 10.1111/j.1471-0528.1988.tb06506.x

[116] Garcia‐Verdugo I , Tanfin Z , Dallot E , Leroy MJ , Breuiller‐Fouche M . Surfactant protein A signaling pathways in human uterine smooth muscle cells. Biol Reprod 2008; 79: 348–355. 18463356 10.1095/biolreprod.108.068338

[117] Lee DC , Romero R , Kim CJ *et al* Surfactant protein‐A as an anti‐inflammatory component in the amnion: implications for human pregnancy. J Immunol 2010; 184: 6479–6491. 20439915 10.4049/jimmunol.0903867 PMC3103775

[118] Agrawal V , Smart K , Jilling T , Hirsch E . Surfactant protein (SP)‐A suppresses preterm delivery and inflammation via TLR2. PLoS One 2013; 8: e63990. 23700442 10.1371/journal.pone.0063990 PMC3659120

[119] Adams Waldorf KM , Persing D , Novy MJ , Sadowsky DW , Gravett MG . Pretreatment with toll‐like receptor 4 antagonist inhibits lipopolysaccharide‐induced preterm uterine contractility, cytokines, and prostaglandins in rhesus monkeys. Reprod Sci 2008; 15: 121–127. 18187405 10.1177/1933719107310992 PMC2774271

[120] Mian MOR , He Y , Bertagnolli M *et al* TLR (Toll‐Like Receptor) 4 antagonism prevents left ventricular hypertrophy and dysfunction caused by neonatal hyperoxia exposure in rats. Hypertension 2019; 74: 843–853. 31476902 10.1161/HYPERTENSIONAHA.119.13022

[121] Hutchinson MR , Zhang Y , Brown K *et al* Non‐stereoselective reversal of neuropathic pain by naloxone and naltrexone: involvement of toll‐like receptor 4 (TLR4). Eur J Neurosci 2008; 28: 20–29. 18662331 10.1111/j.1460-9568.2008.06321.x PMC2588470

[122] Wang X , Zhang Y , Peng Y *et al* Pharmacological characterization of the opioid inactive isomers (+)‐naltrexone and (+)‐naloxone as antagonists of toll‐like receptor 4. Br J Pharmacol 2016; 173: 856–869. 26603732 10.1111/bph.13394 PMC4761092

[123] Bradberry JC , Raebel MA . Continuous infusion of naloxone in the treatment of narcotic overdose. Drug Intell Clin Pharm 1981; 15: 945–950. 7338189 10.1177/106002808101501205

[124] Hutchinson MR , Shavit Y , Grace PM , Rice KC , Maier SF , Watkins LR . Exploring the neuroimmunopharmacology of opioids: an integrative review of mechanisms of central immune signaling and their implications for opioid analgesia. Pharmacol Rev 2011; 63: 772–810. 21752874 10.1124/pr.110.004135 PMC3141878

[125] Jiang X , Ni Y , Liu T , Zhang M , Ren H , Xu G . Inhibition of LPS‐induced retinal microglia activation by naloxone does not prevent photoreceptor death. Inflammation 2013; 36: 42–52. 22869199 10.1007/s10753-012-9518-6

[126] Wang TY , Su NY , Shih PC , Tsai PS , Huang CJ . Anti‐inflammation effects of naloxone involve phosphoinositide 3‐kinase delta and gamma. J Surg Res 2014; 192: 599–606. 25016442 10.1016/j.jss.2014.06.022

[127] Dailey PA , Brookshire GL , Shnider SM *et al* The effects of naloxone associated with the intrathecal use of morphine in labor. Anesth Analg 1985; 64: 658–666. 3160259

[128] Chin PY , Dorian CL , Hutchinson MR *et al* Novel Toll‐like receptor‐4 antagonist (+)‐naloxone protects mice from inflammation‐induced preterm birth. Sci Rep 2016; 6: 36112. 27819333 10.1038/srep36112 PMC5098167

[129] Wahid HH , Moldenhauer JS , Rice KC , Hutchinson MR , Robertson SA . Platelet activating factor (PAF) induces preterm birth in mice through TLR4‐dependant induction of pro‐inflammatory cytokines. Reprod Sci 2017; 24: 78A Meeting Abstract O‐075.

[130] Chin PY , Dorian C , Sharkey DJ , *et al* Toll‐like receptor‐4 antagonist (+)‐naloxone confers sexually dimorphic protection from inflammation‐induced fetal programming in mice. Endocrinology 2019; 160: 2646–2662. 31504393 10.1210/en.2019-00493 PMC6936318

[131] Eriksson JG , Kajantie E , Osmond C , Thornburg K , Barker DJ . Boys live dangerously in the womb. Am J Hum Biol 2010; 22: 330–335. 19844898 10.1002/ajhb.20995 PMC3923652

[132] Ozanne SE , Constancia M . Mechanisms of disease: the developmental origins of disease and the role of the epigenotype. Nat Clin Pract Endocrinol Metab 2007; 3: 539–546. 17581623 10.1038/ncpendmet0531

[133] Burton GJ , Fowden AL . Review: the placenta and developmental programming: balancing fetal nutrient demands with maternal resource allocation. Placenta 2012; 33(Suppl): S23–S27. 22154688 10.1016/j.placenta.2011.11.013

[134] Cheng W , Li Y , Hou X *et al* HSP60 is involved in the neuroprotective effects of naloxone. Mol Med Rep 2014; 10: 2172–2176. 25051048 10.3892/mmr.2014.2411

[135] McGuire W , Fowlie PW . Naloxone for narcotic exposed newborn infants: systematic review. Arch Dis Child Fetal Neonatal Ed 2003; 88: F308–F311. 12819163 10.1136/fn.88.4.F308 PMC1721582

[136] Debelak K , Morrone WR , O'Grady KE , Jones HE . Buprenorphine + naloxone in the treatment of opioid dependence during pregnancy‐initial patient care and outcome data. Am J Addict 2013; 22: 252–254. 23617867 10.1111/j.1521-0391.2012.12005.x

[137] Ng PY , Ireland DJ , Keelan JA . Drugs to block cytokine signaling for the prevention and treatment of inflammation‐induced preterm birth. Front Immunol 2015; 6: 166. 25941525 10.3389/fimmu.2015.00166 PMC4403506

[138] Pawelczyk E , Nowicki BJ , Izban MG *et al* Spontaneous preterm labor is associated with an increase in the proinflammatory signal transducer TLR4 receptor on maternal blood monocytes. BMC Pregnancy Childbirth 2010; 10: 66. 20964862 10.1186/1471-2393-10-66 PMC2972234

[139] Kim J , Ko Y , Kwon K *et al* Analysis of monocyte subsets and toll‐like receptor 4 expression in peripheral blood monocytes of women in preterm labor. J Reprod Immunol 2012; 94: 190–195. 22440522 10.1016/j.jri.2012.02.002

[140] Lorenz E , Hallman M , Marttila R , Haataja R , Schwartz DA . Association between the Asp299Gly polymorphisms in the Toll‐like receptor 4 and premature births in the Finnish population. Pediatr Res 2002; 52: 373–376. 12193670 10.1203/00006450-200209000-00011

[141] Rey G , Skowronek F , Alciaturi J , Alonso J , Bertoni B , Sapiro R . Toll receptor 4 Asp299Gly polymorphism and its association with preterm birth and premature rupture of membranes in a South American population. Mol Hum Reprod 2008; 14: 555–559. 18723631 10.1093/molehr/gan049 PMC2547094

[142] Elovitz MA , Brown AG , Breen K , Anton L , Maubert M , Burd I . Intrauterine inflammation, insufficient to induce parturition, still evokes fetal and neonatal brain injury. Int J Dev Neurosci 2011; 29: 663–671. 21382466 10.1016/j.ijdevneu.2011.02.011 PMC3140629

[143] Challis JR , Sloboda DM , Alfaidy N *et al* Prostaglandins and mechanisms of preterm birth. Reproduction 2002; 124: 1–17. 12090913 10.1530/rep.0.1240001

[144] Kemp MW , Saito M , Newnham JP , Nitsos I , Okamura K , Kallapur SG . Preterm birth, infection, and inflammation advances from the study of animal models. Reprod Sci 2010; 17: 619–628. 20581349 10.1177/1933719110373148

